# Fast and Accurate Ring Strain Energy Predictions with Machine Learning and Application in Strain-Promoted Reactions

**DOI:** 10.1021/jacsau.5c00667

**Published:** 2025-10-13

**Authors:** Zhen Liu, Jessica Vinskus, Yue Fu, Peng Liu, Kevin J. T. Noonan, Olexandr Isayev

**Affiliations:** † Department of Chemistry, 6612 Carnegie Mellon University, Pittsburgh, Pennsylvania 15213, United States; ‡ Modeling and Informatics, 2793 Merck & Co., Inc., Rahway, New Jersey 07065, United States; § Department of Chemistry, 6614 University of Pittsburgh, Pittsburgh, Pennsylvania 15260, United States

**Keywords:** ring strain energy, machine learning, homodesmotic reaction, click chemistry, polymerization, DFT

## Abstract

Ring strain energy (RSE) is crucial for understanding molecular reactivity, with broad implications in polymerization, click chemistry, drug discovery and beyond. However, quantitatively determining RSE through experiments or quantum mechanics (QM) is resource-intensive, limiting its application on a large scale. We present a machine learning (ML)-based workflow that enables the reliable and efficient prediction of RSE, entirely bypassing traditional QM calculations. Our workflow employs AIMNet2 machine learning interatomic potentials and Auto3D for the identification of low-energy conformers and RSE computation. Remarkably, it achieves an *R* ^2^ of 0.997 and a mean absolute error (MAE) of 0.896 kcal/mol when benchmarked against the ωB97M-D4/Def2-TZVPP method, while running orders of magnitude faster than DFT calculations. To demonstrate the utility of our workflow, we successfully differentiated reactive from nonreactive molecules in copper-free click chemistry, [3 + 2] cycloaddition reaction and ring-opening metathesis polymerization, underscoring its transferability to diverse molecular systems. Additionally, we compiled the RSE Atlas, a computational database encompassing 16,905 single-ring molecules, offering a valuable resource for investigating factors influencing RSE. Our approach transforms RSE into a readily computable property, facilitating its integration into reaction designs.

## Graphical abstract

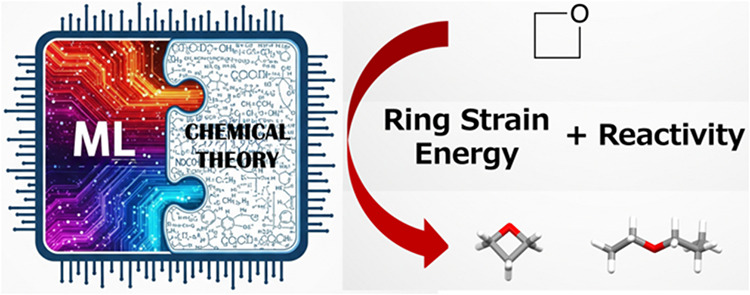

## Introduction

Ring strain energy (RSE) is one of the few concepts that bridges the interest of both experimental and computational chemists.^1^ Since Adolf von Baeyer introduced the concept of “strain theory” in 1885,^2^ RSE has been extensively studied as a means to understand and predict molecular reactivity through both experimental measurements and computational modeling. When atoms form a ring, the bond angles deviate from the natural bond angles found in acyclic molecules. This deviation in geometry leads to a change in molecular energy, which can be quantified by the RSE.^3^ Beyond its fundamental importance in advancing theoretical methodologies, RSE plays a pivotal role in a wide range of fields, including organic synthesis, material design and drug discovery.^4–6^

Experimental chemists leverage RSE in molecular design. Strained molecules exist widely in nature, and provide unique challenges and unexpected opportunities for the development of new reactions and strategies.^7–9^ The release of RSE is a significant driving force for many chemical reactions and the design of novel synthesis methodologies.^10–12^ Highly strained molecules, such as cyclopropene derivatives, can be utilized for synthesizing well-controlled and sequence-regulated polymers by adjusting the ring strain through modulation of substituents.^13^ Less strained molecules, like cyclooctyne derivatives, can be tuned for multiple copper-free click reactions.^14,15^ Additionally, unusual ring structures, such as 1,2,3-cyclohexatriene and its derivatives, can engage in a variety of reaction modes, enabling the novel synthesis of complex molecules.^16^

Computational chemists strive to quantify RSE. Generally, RSE can be determined from the energy difference between the cyclic molecule and an appropriate reference counterpart. While this may seem straightforward, selecting a suitable reference is not well-defined, let alone the methods for accurately obtaining its heat of formation (Figure 1). A common approach is to identify the equivalent groups for each heavy atom in the cyclic molecule, then the RSE is computed as the difference in heat of formation of the cyclic molecule and the equivalent groups. The heat of formation of equivalent groups can be obtained by regression analysis of many organic compounds or using the additivity rules. However, this approach becomes ambiguous for complex molecules.^17,18^ Alternatively, theoretical estimation of RSE can be achieved via ring bond angles and ring bending force constants, i.e.,

$$\mathrm{RSE}=\sum_{i=1}^{N}k_{i}^{\theta }{\left(\theta_{i}-\theta_{i}^{0}\right)}^{2}$$

where θ_*i*_ and θ_*i*_^0^ are the bond angles in the cyclic and strain-free species, respectively, and *k*_*i*_^θ^ is the force constant associated with the bond angle. However, accurately quantifying the ring bending force constants is nontrivial.^19^

**Figure 1:**
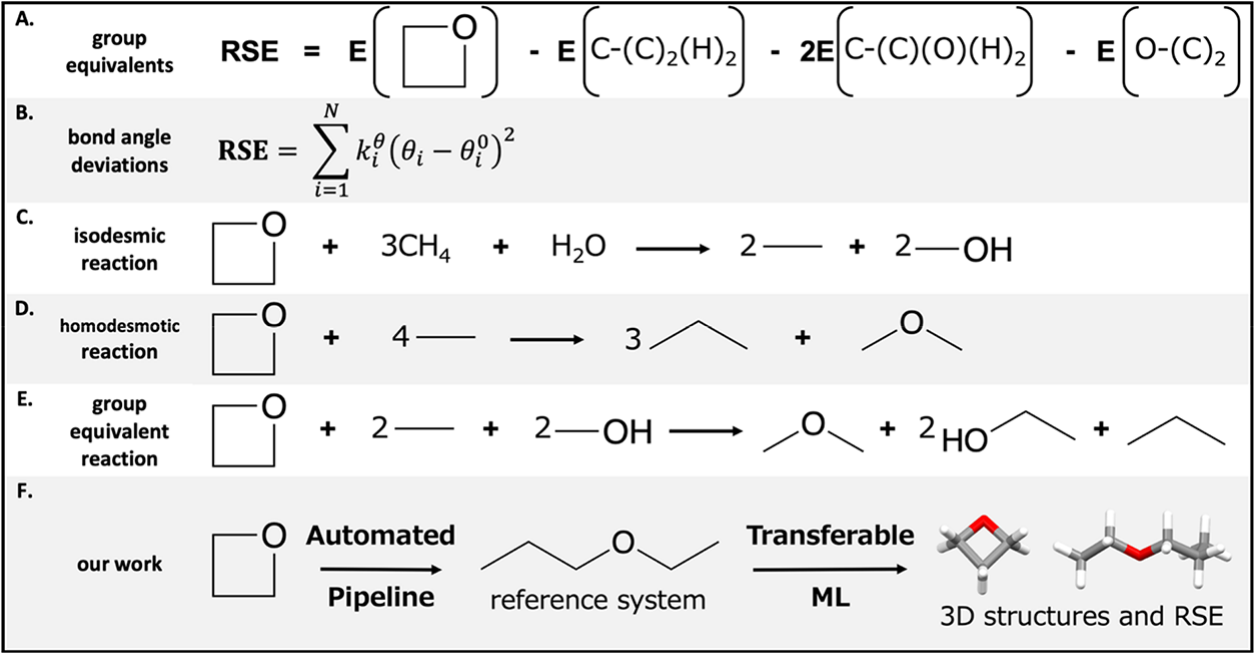
Methods for quantifying RSE. (A) Group equivalents: C-(C)_2_(H)_2_ denotes a carbon atom bonded to 2 carbon atoms and 2 hydrogen atoms. (B) Comparison of bond angles in a cyclic molecule versus a suitable reference system. θ_*i*_ and θ_*i*_^0^ denote bond angles in a cyclic molecule and the corresponding reference system, respectively. *k*_*i*_^θ^ denotes the force constant. (C) Isodesmic reaction. (D) Homodesmotic reaction. (E) Group equivalent reaction. (F) This work.

RSE can also be calculated using hypothetical reactions where simple molecules are used as references and to balance the equation. In these reactions, the reactant is the strained molecule, and product is the unstrained reference. One simply needs to compute the energies of all species in the equation, determine the reaction energy, and the RSE is at hand. There are three variations: the isodesmic reaction, the homodesmotic reaction and the group equivalent reaction. The isodesmic reaction conserves the number of bonds of a given formal type. While it is simple to define, local atom environment is not taken into considerations.^20^ The homodesmotic reaction conserves the heavy atoms, considering both the hybridization states and number of hydrogen atoms on heavy atoms. The homodesmotic reaction is a significant improvement compared with the isodesmic reaction.^21^ Lastly, the group equivalent reaction pairs each equivalent group in the cyclic molecule with an equivalent group in a short acyclic molecule. The group equivalent reaction effectively conserves the next-nearest neighbors.^22^ In all reaction-based methods, it is necessary to find the optimal 3D structure, compute the energies, and then determine the RSE as the energy difference.^3^ This process requires extensive human involvement in designing the reaction and intensive calculations, hindering the widespread application of RSE in reaction design.^23^

Recent developments in machine learning (ML) provide efficient and accurate alternatives for computing RSE. Machine learning interatomic potentials (MLIPs), such as AIMNet2 and ANI, have achieved chemical accuracy in just a fraction of the time required for QM methods.^24–27^ The Auto3D package, supported by the AIMNet2 and ANI models as the backend, can reliably retrieve low-energy conformers and compute thermodynamic properties.^28–30^ Furthermore, the expansion of general ML data and methodologies has enabled the modeling of diverse molecular properties.^31–35^

In this work, we developed the *AIMNet2 RSE workflow* for predicting RSE, fully bypassing QM calculations. AIMNet2 is a pretrained transferable ML potential that includes explicit long-range electrostatics and dispersion. It is applicable to neutral and charged states and covers the chemistry space with 14 chemical elements (all nonmetals).^27^ The workflow integrates ML-based energy predictions with classical chemical theory, enabling efficient, accurate, and explainable RSE computations. It automatically constructs homodesmotic equations based on established chemical principles, incorporating Auto3D for conformer generation and AIMNet2 for energy evaluation. Compared to purely data-driven models, our approach provides detailed information on intermediate steps—such as optimized conformers and molecular energies of species in the homodesmotic reaction—offering valuable insight into the origins of ring strain and allowing better extrapolation by leveraging chemical knowledge as an inductive bias.

In contrast to traditional computational chemistry methods, the workflow fully bypasses QM calculations, substantially accelerating RSE evaluation. Using this framework, we assessed RSE across a wide range of reactants and demonstrated its utility as a univariate descriptor for distinguishing reactive from unreactive molecules in click chemistry and ring-opening metathesis polymerization (ROMP) reactions. In addition, we constructed an interactive RSE Atlas comprising 16,905 single-ring molecules, providing a valuable resource for understanding RSE trends and informing experimental design. The AIMNet2 RSE workflow transforms RSE into a readily computable property suitable for large-scale screening and real-time applications, thereby facilitating a range of research and discovery efforts.

## Results and Discussion

### AIMNet2 RSE Workflow

The AIMNet2 RSE workflow comprises three major components: constructing the nonstrained reference counterpart, obtaining reliable 3D structures and computing the RSE as the energy difference (Figure 2). Given a molecule, the workflow first detects the atoms forming a ring. For each ring, a single carbon–carbon (C–C) bond is broken and each end is appended to a methyl group. The number of substituents on the bonding atoms and the neighbors around the bounding atoms are considered when selecting a bond to be broken. There are three scenarios. In the first scenario, the workflow breaks an unsubstituted C–C bond that is not connected to heteroatoms or functional groups. This is called “ideal breaking”. In the second scenario, where no ideal bond is available, the workflow considers any unsubstituted C–C bond. This is called “relaxed breaking”. In the third scenario, where no unsubstituted C–C bond is available, the workflow considers any available C–C bond of the ring. This is called “forced breaking”. In the first and second scenarios, if multiple valid bonds are present, one of the valid bonds is randomly broken. The RSE difference resulted from breaking different bonds is typically within 1 kcal/mol in these scenarios. For the third scenario, the bond whose resulting counterpart has the lowest energy is broken. The RSE difference obtained by breaking different bonds in each scenario is detailed in Figures S1–S3.

**Figure 2:**
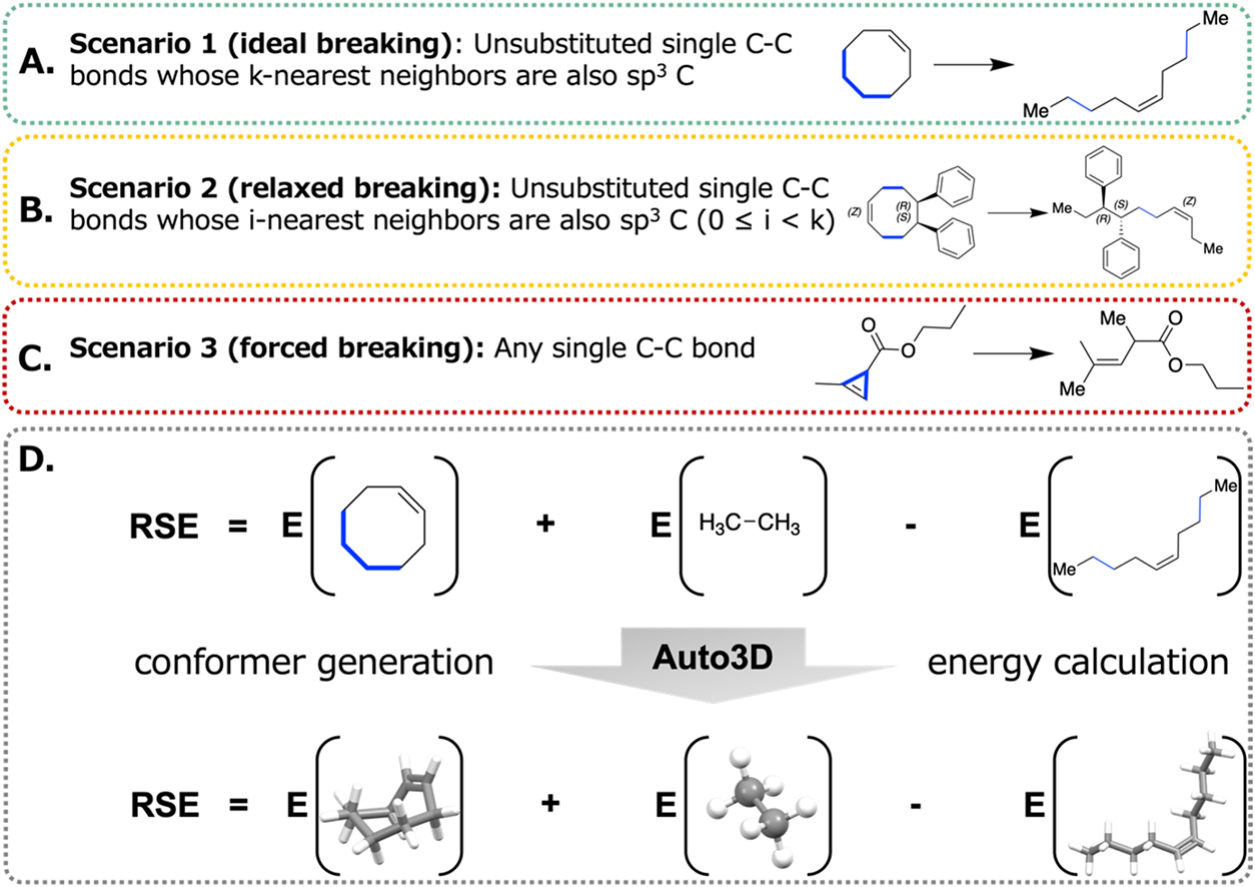
Overview of the AIMNet2 RSE workflow. (A) Ideal bond-breaking: a bond is considered “ideal” if its k-nearest neighboring bonds (default *k* = 1) are also unsubstituted single C–C bonds. (B) Relaxed bond-breaking: with *i* = 0 (default), any unsubstituted single C–C bond can be considered, regardless of neighboring context. (C) Forced breaking: if no unsubstituted C–C bond is present, the workflow evaluates all C–C bonds and selects the one yielding the lowest-energy reference. (D) The homodesmotic equation for computing RSE. For each molecule in the homodesmotic equation, Auto3D is used to get the low-energy conformer and energy.

Using a ring structure and its reference counterpart, a homodesmotic reaction is constructed to calculate the RSE. In the homodesmotic reaction, the ring is on the left side, and the broken ring is on the right side. Ethane is added to ensure that the number of atoms and bond types are equal on both sides of the reaction. The RSE is calculated as the energy of the ring side minus the energy of the broken ring side.

For each molecule in the homodesmotic reaction, Auto3D^28^ is used to identify the low-energy conformer and compute the energies. Auto3D locates the low-energy conformers by enumerating conformer candidates, optimizing the ensemble and selecting those with the lowest energies. In the default Auto3D configuration, the isomer engine is RDKit^36^ and the optimization engine is AIMNet2.^27^ The AIMNet2 model is transferable, allowing it to be applied to new molecules without additional training, similar to conventional computational chemistry tools. This capability enables a highly efficient RSE calculation process. Unlike other ML methods that require a training set and struggle to generalize beyond it, AIMNet2 offers a distinct advantage in flexibility and applicability. Unless otherwise noted, the RSE in this work is reported in terms of enthalpy to be consistent with the historical experimental methods that determine RSE via heat of formation.^23^

### Method Validation

As a proof of concept, we applied the AIMNet2 RSE workflow to compute RSE values for a series of ring systems and compared the results with established literature values. As shown in Figure 3, the AIMNet2 RSE workflow exhibits strong consistency with experimental conventional ring strain energies (CRSE).^1^ CRSEs are derived from the difference between the experimental heat of formation of a cyclic compound and that of a corresponding strain-free reference. The strain-free reference energies were estimated using the group equivalent method, in which the energy of each group is obtained as an average based on regression analysis of a large set of organic molecules.

**Figure 3:**
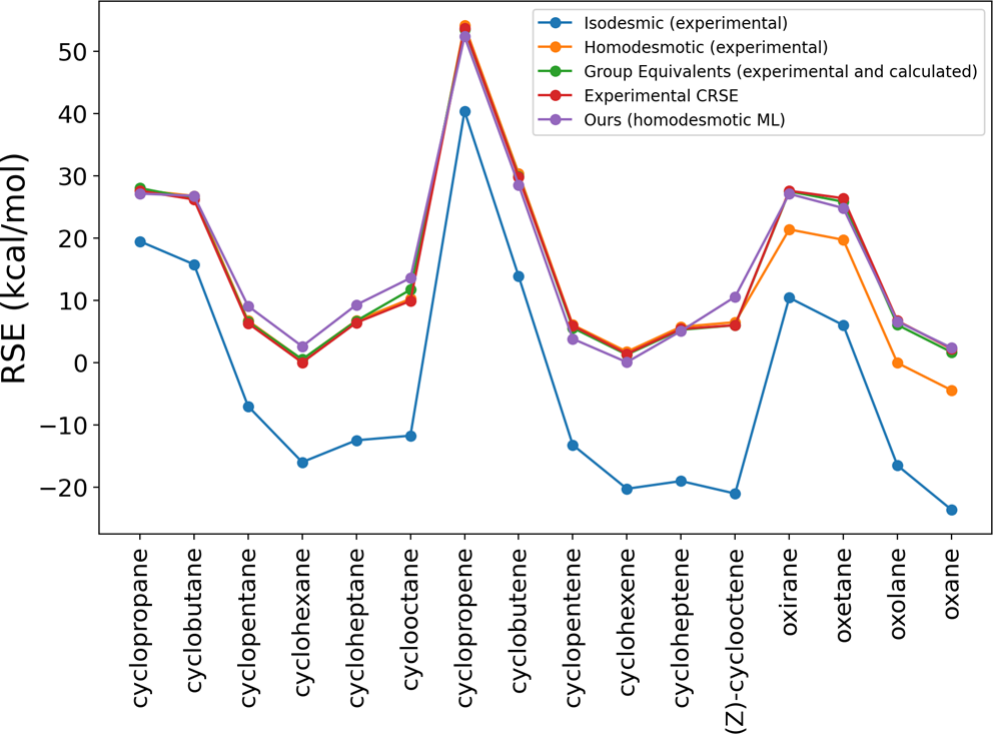
RSE calculated using different methods. The RSE values are sourced from Table 3.21 of Reference.^1^ For the isodesmic and homodesmotic methods, experimental heats of formation were used to construct the corresponding equations. The group equivalent method primarily relied on experimental values, supplementing them with computational estimates when experimental data were unavailable. The experimental CRSE values are derived purely from experimental data. Our method computes RSEs using homodesmotic-like transformations, with energies predicted by the AIMNet2 model.

For comparison, RSEs computed from other established methods are also included (Table 1). These approaches typically rely on experimentally or theoretically determined heats of formation for simpler molecules, and construct isodesmic, homodesmotic, or group equivalent reactions to estimate RSE. Among them, the group equivalent method achieved the lowest mean absolute error (MAE) and the highest Spearman correlation with CRSE. Our ML-based workflow performed slightly better than the homodesmotic approach, and both methods showed close agreement with CRSE values. While RSE values from isodesmic reactions follow a similar trend, they are systematically lower than CRSEs.

**Table 1:** Comparing Different Methods to the Experimental CRSE

	isodesmic	homodesmotic	group equivalent	ours
MAE (kcal/mol)	18.55	1.94	0.38	1.64
Spearman correlation	0.86	0.89	0.99	0.96

It is worth noting that our method is entirely ML-based, enabling rapid RSE predictions while maintaining high fidelity. These results demonstrate that the workflow can reliably construct homodesmotic-like transformations and accurately estimate RSEs.

We further validated the accuracy of the AIMNet2 RSE workflow with 20 extensively studied polymerizable ring systems^13,37^ (Figure 4). These 20 molecules can be categorized into 5 groups: cyclopropene derivatives, cyclopentane derivatives, cycloheptene derivatives, cyclooctane derivatives and a fused ring. While these systems are commonly studied in polymer research, their RSE values are not readily available in the literature. Therefore, we compared the RSE values calculated with our method and a QM method.

**Figure 4:**
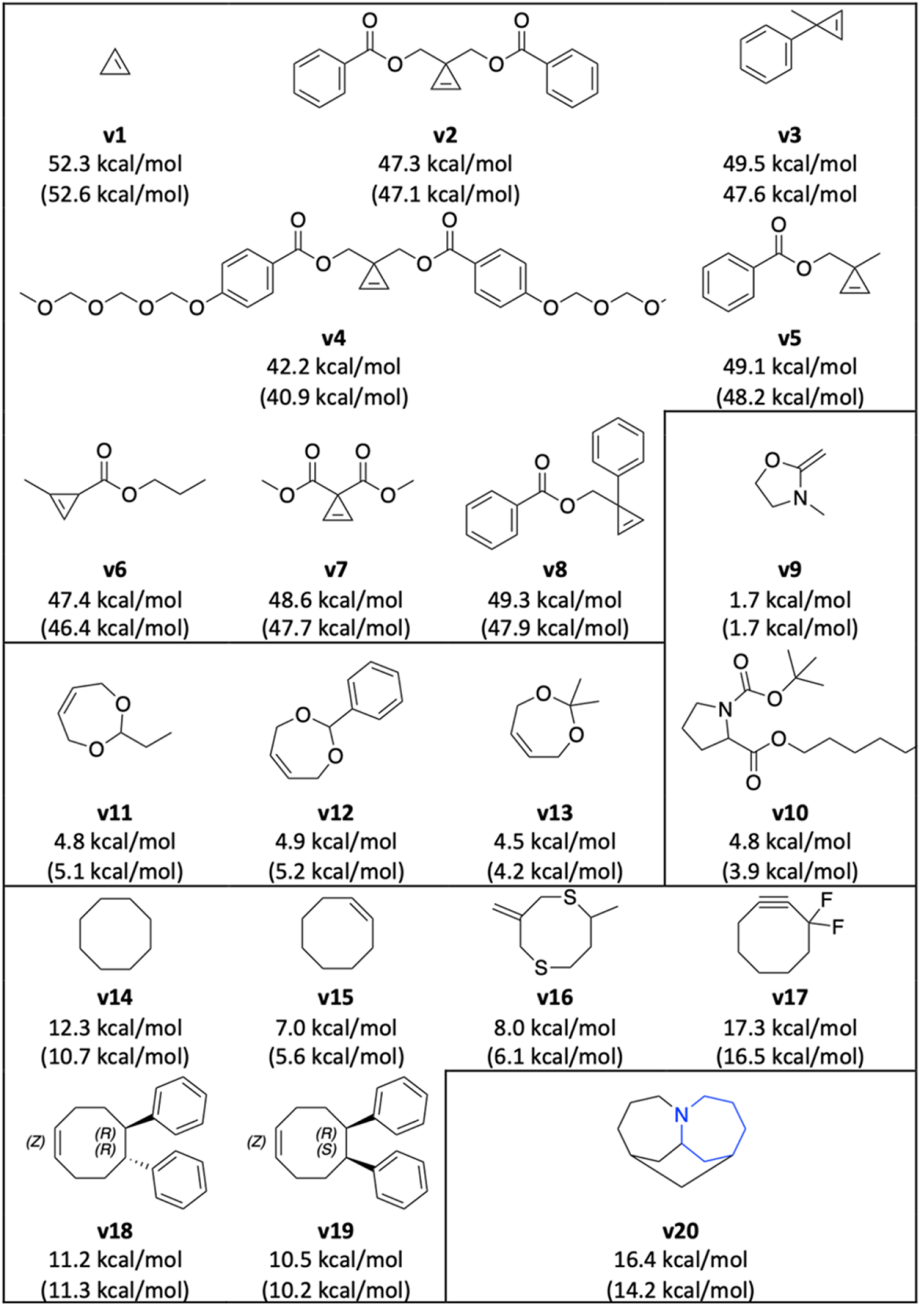
Comparing the RSE calculated using AIMNet2 (top) and ωB97M-D4/Def2-TZVPP (bottom, in parentheses). For **v20**, we measured the RSE release by breaking the ring colored in blue.

The AIMNet2 RSE values were compared with those obtained from the corresponding DFT method (ωB97M-D4/Def2-TZVPP, ORCA 5.0.4).^38–40^ The overall MAE was 0.90 kcal/mol, demonstrating the reliability of the workflow for calculating RSE. A scatter plot is attached in Figure S4. Notably, the computational efficiency of the AIMNet2 RSE workflow is significantly higher than that of traditional DFT calculations. On a single NVIDIA RTX 3090 GPU, the workflow required approximately 30 min to evaluate the RSEs, whereas the DFT approach required around 500 CPU hours. This comparison highlights the substantial speed advantage of our method—achieving a reduction in computational cost by several orders of magnitude while maintaining acceptable accuracy.

Five-membered rings generally exhibit lower ring strain energies (RSE) compared to other ring sizes, consistent with the notion that their internal bond angles closely approximate those found in acyclic systems. By contrast, cyclopropene derivatives typically possess some of the highest RSE values due to severe angle strain and torsional repulsion. The introduction of electron-withdrawing or donating substituents to the C3 positions of cyclopropene notably affects the RSE. Here, the RSE is affected by hyperconjugative aromaticity, whereby the electron density of the σ/σ* orbitals affects the aromaticity and stability of the planar cyclopropene ring.^41^

### Copper-Free Click Chemistry

To illustrate the practical value, we explored the potential to screen reactive molecules with the AIMNet2 RSE workflow. Our investigation focused on the 1,3-dipolar cycloaddition of cyclooctynes with azides, a key reaction in ″copper-free click chemistry″ widely applied in drug design.^42–45^ The release of RSE in cyclooctynes serves as a driving force in this reaction, making the effective computation of RSE valuable for predicting cyclooctyne reactivity and guiding rational molecule design. We selected cyclooctyne derivatives to validate the correlation between RSE and reactivity. The reactivity of cyclooctyne derivatives was characterized by experimental second-order rate constants, sourced from several experimental reports.^43,46–52^ The list of cyclooctyne derivatives is available in the Figure S5.

The homodesmotic equation for characterizing the strain release is defined as shown in Figure 5A. Butene and butyne are used to balance the number of atoms in the homodesmotic equation. The energy difference between the two sides of the homodesmotic reaction can be thought of as a partial ring strain energy release during the 1,3-dipolar cycloaddition of cyclooctynes with azides. The click reaction reduces the triple bond in cyclooctyne to a double bond instead of breaking the ring, so the homodesmotic equation for computing ring strain release is different from conventional homodesmotic reaction where a ring is broken.

**Figure 5:**
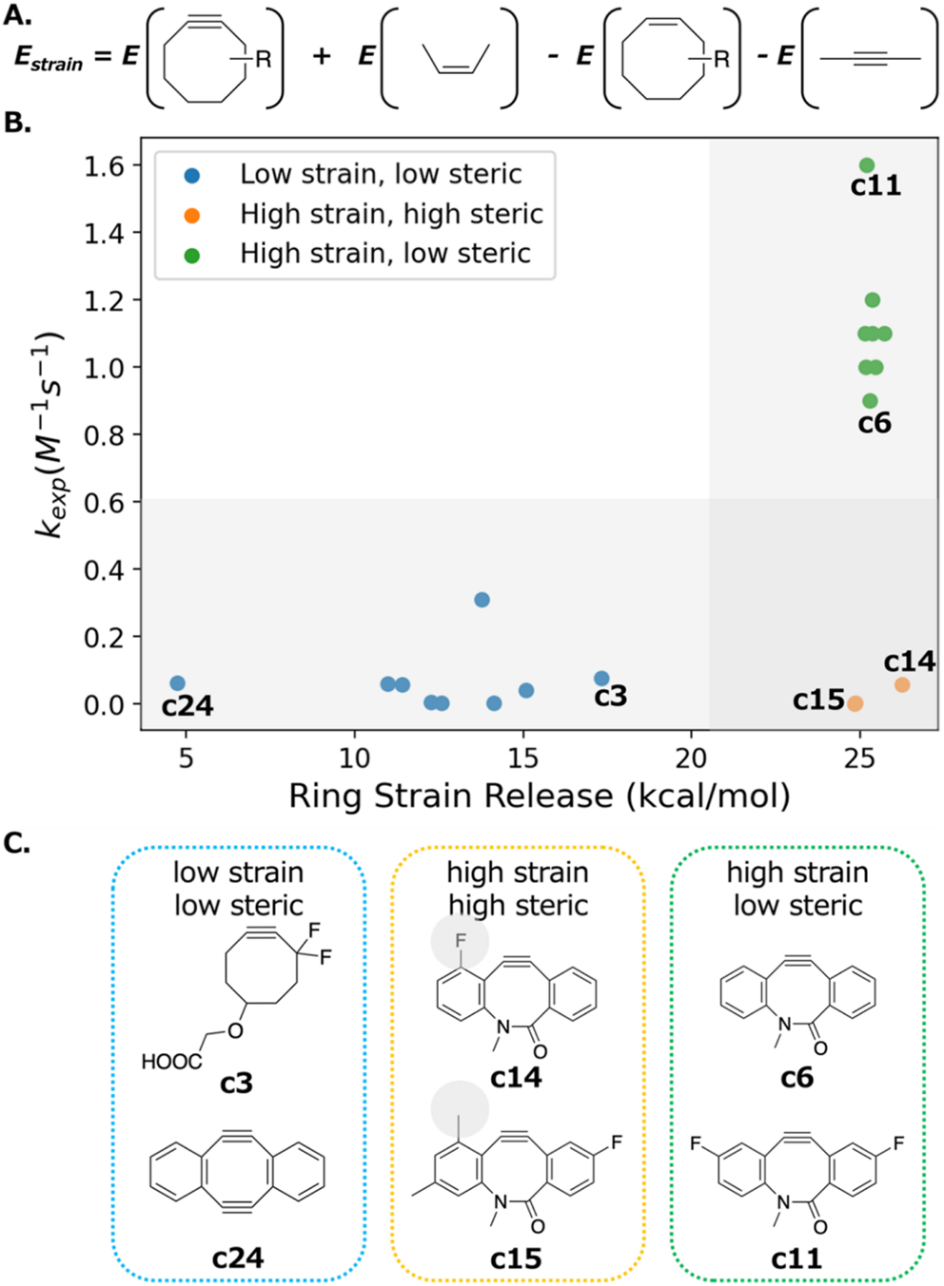
Strain release captures the reactivity cliff in click chemistry. (A) The homodesmotic reaction for computing ring strain release. (B) The relationship between the second-order reaction rate constant and the ring strain release. (C) Example molecules for each group.

We computed the RSE for 21 cyclooctyne derivatives, of which the experimental second-order reaction constant rates were summarized by Bertozzi et al.^14^ Figure 5B shows the relationship between the RSE and molecular reactivity. When the RSE is below a certain threshold, the molecules react slowly, as indicated by the small rate constants. This corresponds to the molecules represented by blue dots at the bottom left of the figure. When the RSE is high, the molecule is reactive if other conditions are also favorable. This corresponds to the molecules represented by green dots at the top right of the figure. There is at least 5 kcal/mol gap in the ring strain release between the reactive cyclooctyne derivatives (green dots) and the nonreactive cyclooctyne derivatives (blue dots).

If there is sufficient ring strain release but other conditions are not met, the cyclooctyne derivatives remain low in reactivity. This scenario is represented by orange dots at the bottom right of the figure. For example, molecule **c14** has adequate strain release (26.3 kcal/mol) to potentially react, but the fluorine atom near the triple bond introduces steric hindrance that prevents the approach of azides. We provided two examples for each scenario in Figure 5C. The specific values for ring strain release and rate constants are recorded in Table S1.

Cyclic alkynes are widely used in strain-driven reactions, and understanding the trends in their reactivity remains an active area of research. In general, strain, electronic effects, orbital interactions, and HOMO–LUMO gaps are interrelated and jointly shape reactivity.^53,54^ Nevertheless, RSE effectively captures the reactivity cliff observed in this class of reactions. Given the efficiency of the AIMNet2 RSE workflow in computing RSE, it provides a practical and predictive univariate criterion for identifying reactive cyclooctyne derivatives, with broad potential utility in reaction design.

### [3 + 2] Cycloaddition Reactions

The proposed RSE workflow was further evaluated on a broader set of [3 + 2] cycloaddition reactions, which are of significant importance in biochemistry. Our goal was to demonstrate the workflow’s applicability across a wide range of molecules and to highlight the value of computed RSE in identifying promising reactants. To this end, we benchmarked our workflow against a computational reaction profile database for [3 + 2] cycloadditions. This database includes transition states, activation energies (Δ*G* ^‡^), and reaction energies (Δ*G*_r_), calculated at the B3LYP-D3­(BJ)/def2-TZVP//B3LYP-D3­(BJ)/def2-SVP level of theory. The data set comprises 1516 dipoles and 713 dipolarophiles, yielding over 5,000 unique reactions. The data set details can be found in the original publication.^55^

From this data set, we selected only nonaromatic single-ring molecules for RSE computation, resulting in 1319 structures. The entire RSE computation process was fully automated by our workflow. In some cases, RDKit failed to generate initial conformers due to complex ring geometries. Additionally, a subset of structures did not converge during ML-based geometry optimization or free energy estimation. The AIMNet2 RSE workflow offers multiple settings for RSE computation. In practice, we found that using OpenEye Omega as the isomer engine and increasing the maximum number of optimization steps significantly reduced failure rates. However, the results presented here were obtained using the default settings: RDKit as the isomer engine and moderate optimization parameters, selected to reflect the computational infrastructure available to typical users. Under these conditions, we successfully obtained RSE values for 371 products and 170 dipolarophiles. We then examined the correlation between RSE and the reaction profiles.

The RSE distributions for products and dipolarophiles are shown in Figure 6A. The mean RSE for dipolarophiles is 18.90 kcal/mol, while that for products is 2.43 kcal/mol. This indicates that dipolarophiles generally possess higher RSE than the resulting products, suggesting that the reaction process typically involves the release of ring strain. Moreover, dipolarophiles with higher RSE are likely to participate in more favorable reactions. This trend is further supported in Figure 6B, where reactions grouped by dipolarophile RSE show lower reaction energies when the dipolarophile RSE is higher.

**Figure 6:**
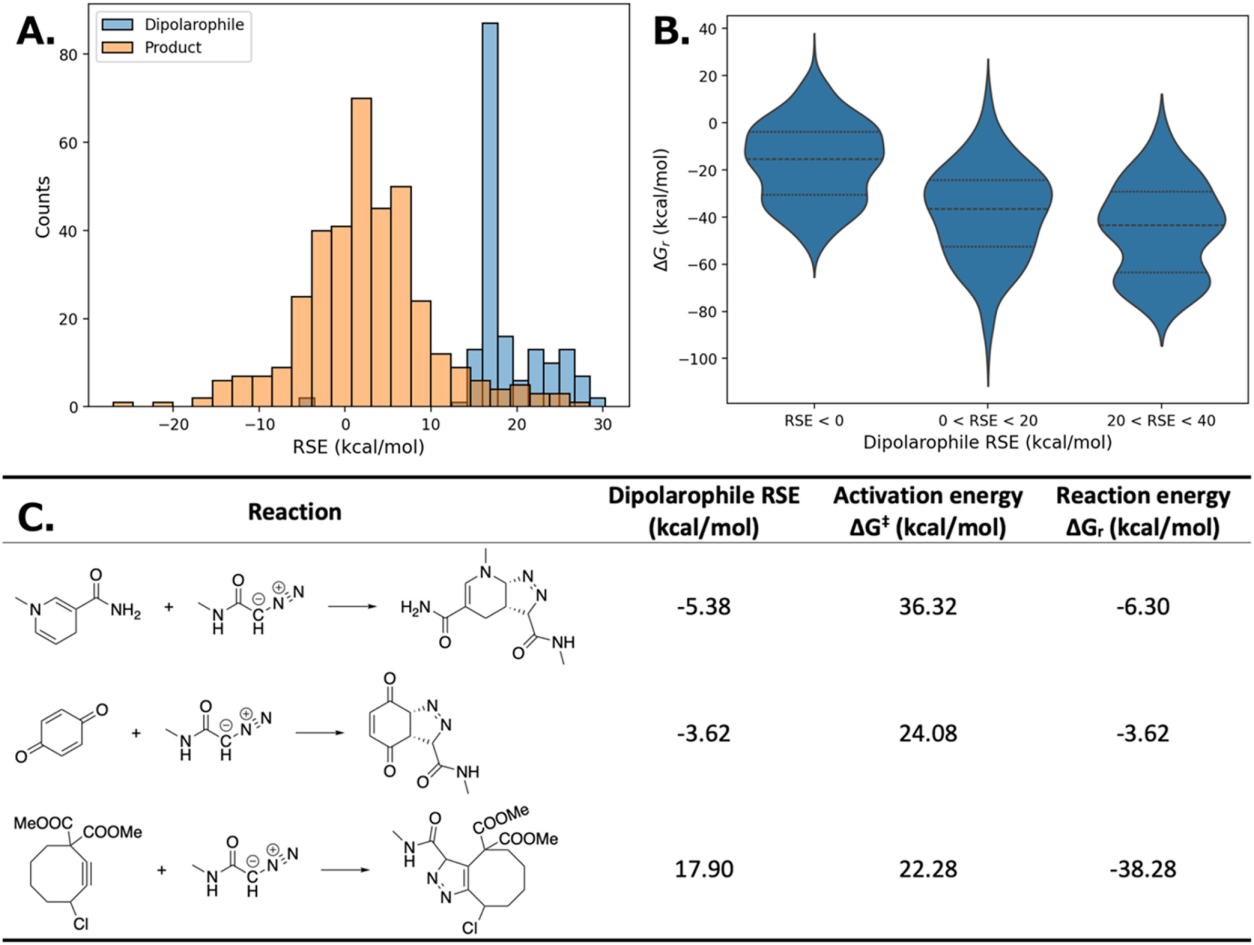
Relationship between RSE and reaction energies in [3 + 2] cycloaddition reactions. (A) RSE distribution for dipolarophiles and products. (B) Reaction energy distribution grouped by dipolarophile RSE. (C) Representative examples showing how RSE modulates both activation energy and reaction energy.

Figure 6C presents example reactions to illustrate how increasing dipolarophile RSE influences reaction energetics. In these examples, the dipole remains constant while the dipolarophile RSE increases from the first to the third reaction. Both the activation energy and reaction energy decrease accordingly, indicating that the reactions become more favorable. These findings align with the original data set’s observation that strained reactants tend to exhibit lower reaction barriers than unstrained counterparts. By enabling fast, large-scale RSE computation, the AIMNet2 RSE workflow provides valuable insights into the role of ring strain in reactivity and offers a useful tool for the design of biorthogonal reactions.

### Ring Opening Metathesis Polymerization (ROMP)

In another case study, we conducted experiments to demonstrate the application of the AIMNet2 RSE workflow for ring-opening metathesis polymerization (ROMP). RSE is a crucial thermodynamic driving force for this reaction. Accurate computation of RSE could allow us to select monomers that are most likely to polymerize effectively. The ROMP and the homodesmotic equation for computing RSE are depicted in Figure 7A,B, respectively. This homodesmotic equation involves breaking the ring at the double bond to mimic the strain release during the ROMP reaction.

**Figure 7:**
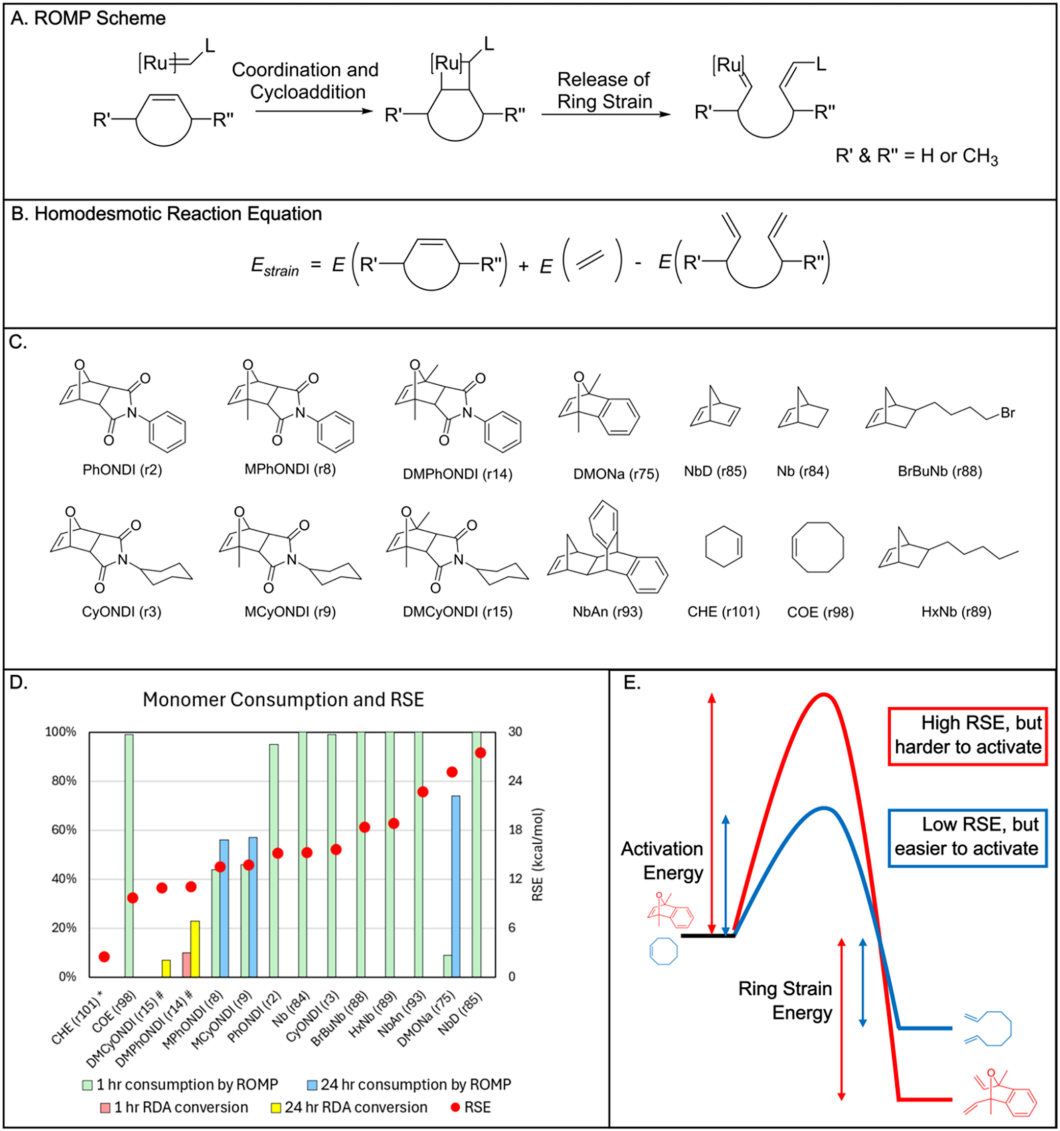
Guide ROMP design with RSE. (A) General scheme for ROMP. (B) Homodesmotic reaction equation for RSE calculation. (C) Monomers used for ROMP experiments. (D) Plot of RSE and monomer consumption. Calculated RSE values are represented by red dots, while monomer consumption is shown as vertical bars. Monomers are consumed via either ROMP (green and blue bars) or the retro-Diels–Alder reaction (RDA, red and yellow bars). For cases where the 1-h consumption was less than 90%, an additional measurement was taken at 24 h. (*) Monomer loss observed due to evaporation; (#) Monomer loss due to the retro-Diels–Alder reaction. (E) Hypothetical reaction coordinate diagrams for the two types of monomers that deviate from the observed trend.

The workflow was used to calculate strain of 107 cyclic alkene monomers. The structures and strain energies are detailed in Figure S6. Mostly norbornene and oxanorbornene derivatives were examined, as these are commonly explored in ruthenium-catalyzed ROMP.^56–58^ These were compared against commercially available monocyclic alkenes as control compounds.^59–65^ The oxanorbornenediimides (ONDIs), derived from cycloaddition between furan and maleimide derivatives were of particular interest since the resultant polymers have been examined as gas transport membranes.^66^ We examined how RSE changes with substitution pattern on the bicyclic rings and in particular, how substituents at the 1 and 4 positions on the ring influence reactivity in Ru-catalyzed ROMP since substituents at these positions can improve photostability of the resultant polyalkenamer.^67,68^

The addition of methyl substituents to the bridgehead carbons (1 and 4 positions) of the norbornene and oxanorbornene derivatives generally leads to a decrease in ring strain, however the effects of a lone substituent are mixed, in most derivatives there is at least a slight decrease (1–2 kcal/mol) of ring strain with the addition of a single group, however the addition of another drops the ring strain a total of 3–5 kcal/mol. Notably the RSE of the furan-cyclohexyl maleimide cycloadduct decreases by 5 kcal/mol with addition of methyl groups at the bridgehead carbons. A similar observation was made for the norbornene derivatives where methyl substituents on the bridgehead decrease the ring strain by 5 kcal/mol. When the substituents are changed, there is a difference in how the RSE is affected. The furan and cyclohexyl maleimide cycloadduct shows an increase of 1 kcal/mol in RSE when one trifluoromethyl group is added, however when the molecule is substituted with two of these groups, the ring strain drops down to 1.5 kcal/mol less than the unsubstituted. This effect changes when it is modeled for the norbornene adduct, which instead shows a drop of 1 kcal/mol for one trifluoromethyl group and a total decrease of 4.5 kcal/mol for the doubly substituted molecule compared to the unsubstituted. The difference in the influence of the trifluoromethyl group on these two monomer sets could be due to the presence of lone pairs on oxygen, which allow for greater polarization by the fluorine atoms.

Substituents often decrease the ring strain of the oxanorbornene and norbornene monomers. The parent oxanorbornene for example, derived from the Diels–Alder reaction of furan and ethylene (r76), has a strain of 18.8 kcal/mol which is higher than the furan maleimide adduct (15.7 kcal). The addition of two methyl groups at the bridgehead positions of the parent oxanorbornene (r78) also decrease the strain to 16.2 kcal/mol. Interestingly, the use of benzyne as a dienophile increases the ring strain dramatically, the nonsubstituted oxanorbornene derivative (r73) has a strain of 27.9 kcal/mol and the related dimethyl derivative (r75) decreases in strain by 2.8 kcal/mol. This dramatic increase of RSE in r73 is likely due to the presence of another π bond in the fused ring system.

From the list of 107 monomers, 14 were selected for ROMP using a variation of Grubbs third generation catalyst (Figure S26) to see if any correlation between RSE and reactivity could be made (Figure 7C,D). The polymerizations were all performed with a 200:1 ratio of monomer:catalyst. The polymerizations were all carried out at ∼0.5 M in dry, degassed CH_2_Cl_2_ at 35 °C under a nitrogen atmosphere.

Monomers were selected based on the ability to tune functional groups and potential for gas transport membranes. Cyclooctene (r98) and cyclohexene (r101) were selected as they have been well studied in ROMP for their reactivity and lack thereof, respectfully. The reactions were analyzed for completion by ^1^H NMR (Figures S12–S25).

For the oxanorbornene derivatives, the computed RSE well correlates with the ROMP reaction outcome as shown in Figure 7D. A decrease in ring strain correlates with a decrease in the rate of polymerization as monomer consumption after 1 h was lower in all instances with increasing methyl substitution (e.g., PhONDI to MPhONDI to DMPhONDI and CyONDI to MCyONDI to DMCyONDI). After 24 h, the dimethyl substituted monomers which have lower ring strain (∼11 kcal/mol), had only undergone a retro-Diels–Alder reaction, and no polymer signals were observed in the ^1^H NMR spectrum of an aliquot removed from the reaction mixture (Figures S16 and S17). The cyclo-reversion could be interfering in the polymerization process as the 2,5-dimethylfuran maleimide Diels–Alder reaction is highly reversible at room temperature and has a low activation barrier (36 kcal/mol for one derivative).^69,70^ The monomers with no methyl groups, which have a higher strain of 15 kcal/mol, reached near complete consumption by ROMP in 1 h. The DMONa monomer derived from 2,5-dimethylfuran and benzyne has a high RSE (25.1 kcal/mol) but is kinetically slower to polymerize than most other derivatives and 74% conversion was noted after 24 h (Figure 7E). Nearly all oxanorbornene derivatives with predicted RSEs of >15 kcal/mol exhibited high reactivity in polymerization and were consumed in 1 h.

While the RSE is a key factor in polymerization, it is not the only consideration, as solvent, monomer stereochemistry, steric effect will influence the outcome. Moreover, kinetics and thermodynamics are often not aligned—a higher RSE (a thermodynamic quantity) does not necessarily correspond to a lower reaction barrier (a kinetic quantity). For simpler monomers, ROMP can occur at lower RSE, although monomers with higher RSE generally tend to be more reactive. For example, cyclohexene (COH) and cyclooctene (COE) have predicted ring strain energies of 2.53 and 9.69 kcal/mol, respectively. Consistent with expectation, the cyclooctene is essentially completely consumed at 1 h,^71^ while cyclohexene is not consumed under the polymerization conditions (minor evaporation was noted in the ^1^H NMR spectrum, Figures S24 and S25). Altogether, the case study highlights the benefits of applying this RSE workflow to help design and rationalize reactions, particularly when comparing a related set of derivatives.

### The RSE Atlas

Building on this success, we constructed a database called the “RSE Atlas” containing 16,905 single-ring molecules. Ring systems were extracted from commercially available compounds (Sigma-Aldrich, Enamine, WuXi, MolPort) and literature-reported building blocks and reagents (PubChem, SureChEMBL). These rings represent the majority of known carbocycles and heterocycles, including an extended set of elements like S, P, Se, B, and I. The ring sizes range from 3 to 9.

For the RSE Atlas, we sampled rings with the highest and lowest RSE values, and validated them by computing the RSE using ωB97M-D4/Def2-TZVPP. The workflow demonstrated strong consistency with the DFT method, achieving an *R* ^2^ of 0.99 and an MAE of 1.76 kcal/mol. A scatter plot comparing RSE values from the workflow and the DFT method is included in the Figure S7.

The overall tendency of the RSE is illustrated in Figure 8. Panel A features a parallel coordinates plot where each ring is represented by a line that connects the ring characteristics, such as the ring size and the number of heteroatoms, and is color-coded by the RSE. This visual format enables intuitive inspection of how each structural factor influences the distribution of ring strain.

**Figure 8:**
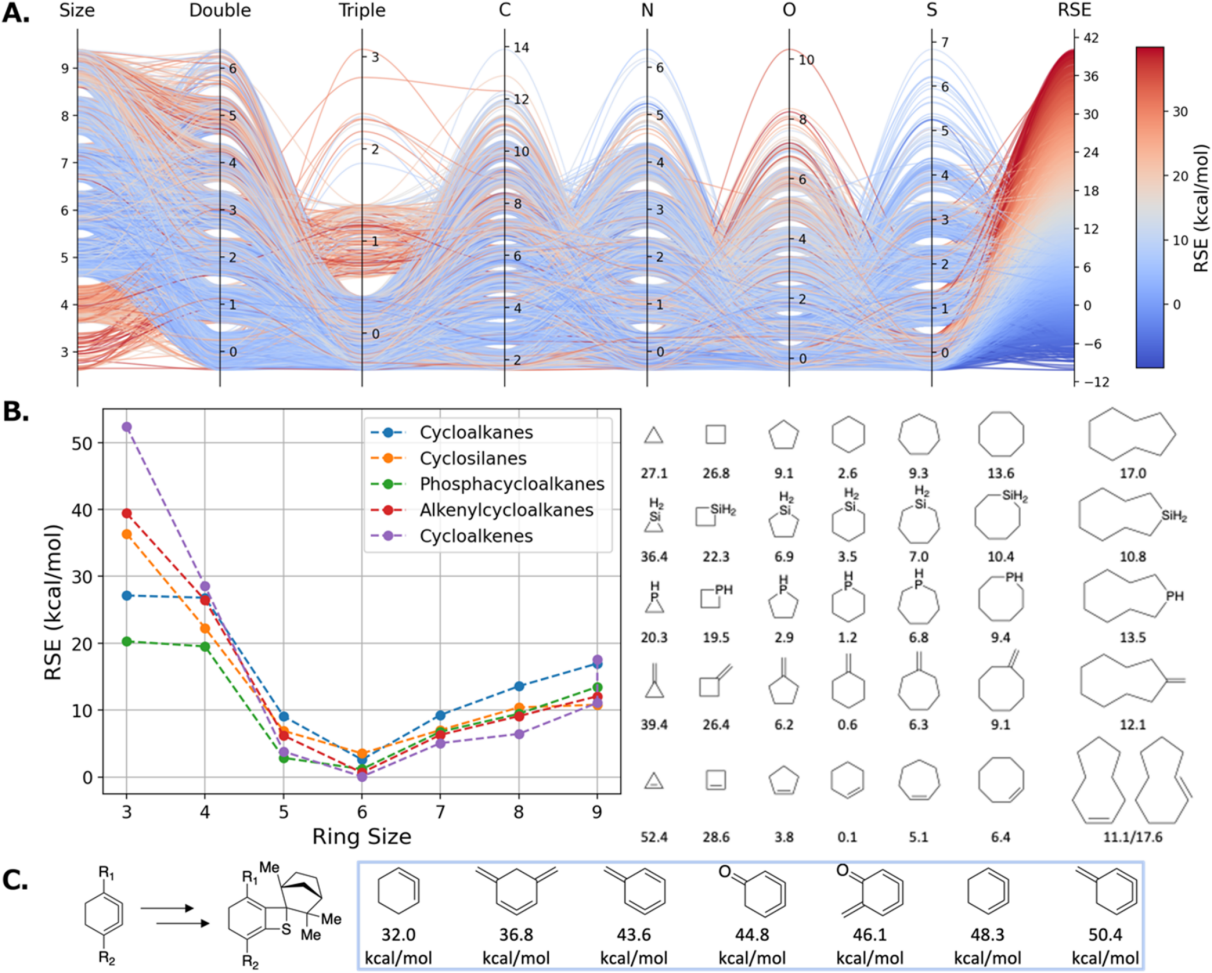
Overview of the RSE Atlas. (A) Parallel coordinate plot for the ring atlas. Each ring is represented with a line, colored by the RSE energy. (B) RSE tendency over the ring size and structure. (C) An example of ring strain-promoted reactions and similar building blocks in the atlas.

A prominent observation from the plot is that five- and six-membered rings tend to exhibit lower RSE values compared to rings with three, four, seven, eight, or nine members. For instance, the axis corresponding to ring size predominantly shows red coloration for sizes 3, 4, and 9, indicating a high prevalence of strained rings in these categories. This trend is also confirmed by the plot in Panel B. The relative stability of five- and six-membered rings is consistent with well-established chemical understanding: these ring sizes possess bond angles that closely approximate the ideal tetrahedral angle of 109.5°, minimizing angle strain. Additionally, these rings can adopt favorable conformations—such as the chair conformation in cyclohexane—which effectively eliminate eclipsing interactions between adjacent atoms and further reduce torsional strain. Medium-sized rings, such as eight- and nine-membered rings, often suffer from transannular strain, which is also observed in the RSE Atlas.

Rings with higher degrees of unsaturation generally display elevated RSE values in comparison to their saturated counterparts. This finding aligns with classical principles of molecular strain, as the incorporation of double or triple bonds introduces bond angles that deviate significantly from the ideal. Moreover, changes in C–H bond and π-bond strengths can contribute to unexpected variations in RSE. For example, cyclobutene exhibits a RSE comparable to that of cyclobutane, primarily due to the strong vinyl C–H bonds and π-bond in cyclobutene.^72^

The influence of heteroatoms on RSE is more nuanced, stemming from differences in bond lengths, bond angles, and lone pair contributions. Bent’s rule provides a useful framework for rationalizing these effects.^73^ In our data set, we observe that rings containing a greater number of oxygen atoms tend to exhibit higher RSE values, whereas those incorporating sulfur atoms often display lower strain. This is likely due to the size difference between the S atom and the O atom. Besides, a recent study also reported a decrease in RSE associated with increased p-character in the atomic orbitals forming endocyclic bonds.^74^

The complete RSE Atlas is accessible via a web application at https://rseatlas.isayevlab.org. Given the breadth of ring structures represented in the atlas, it serves as a valuable resource for identifying strain-promoted building blocks in reaction design. Users can search and filter the data set to explore how specific structural modifications affect RSE, enabling data-driven strategies for the development of novel chemical transformations. For example, the Garg and Hoye groups have recently reported synthetic methodologies that leverage highly strained rings^16,75^ (Figure 8C). Several analogs of these scaffolds are available in the RSE Atlas, offering potential leads for future reaction discovery. Additional statistical analyses of the RSE data set can also be found in Figures S8–S11.

## Conclusions

RSE is important for understanding cyclic molecule reactivity. However, efficiently and accurately computing RSE remains a challenge. Using AIMNet2 machine learning potential and Auto3D, we proposed physics-based ML calculations that predicts RSE with high accuracy and efficiency. Unlike conventional ML methods, it requires no additional training and demonstrates excellent transferability across diverse molecules. Compared to traditional computational chemistry methods, it produces results within minutes. The workflow has been validated both computationally and experimentally. In a benchmark study, the RSE workflow achieved an MAE of around 1 kcal/mol and an *R* ^2^ of 0.997 with respect to the accurate ωB97M-D4/Def2-TZVPP calculations.

Practical case studies showed that our methods could reliably distinguish unreactive molecules from reactive ones. For the copper-free click reactions, the ring strain release gap is around 7 kcal/mol between the reactive cyclooctynes and the nonreactive molecules. For the [3 + 2] cycloaddition reactions, dipolarophiles with higher RSE tend to have more favorable reaction profiles. For the ROMP reactions, norbornene derivatives need around 15 kcal/mol ring strain energy to be thermodynamically favored. These applications demonstrated the transferability and practical value of the RSE workflow.

To empower future strain-controlled reaction design, we compiled a database of 16,905 ring systems. These molecules represent a majority of known single-ring systems from primary literature, patents, commercial building blocks and reagents. The RSE Atlas will be useful for understanding the factors that influence ring strain energy and provides a crucial reference for designing building blocks for various strain-promoted reactions. The AIMNet2 RSE workflow and the RSE Atlas are open-sourced, enhancing their value for future experimental and computational methodology development.

The limitations of the current study are worth noting. First, the current RSE Atlas contains only single-ring molecules. For molecules with multiple rings, it requires human intervention to sequentially apply the workflow to each ring of the molecule until all rings have been exhausted. We provided an example of applying the workflow to compute the RSE for prismane, cubane, and adamantane in Table S3. Due to the integration of a transferable ML model with chemical theories, we observed reasonable performance on these complex systems. For multiring molecules, automatically constructing the homodesmotic reactions is not always trivial, and searching for reasonable conformers—especially for flexible macrocycles—is a research topic worth future investigation. Second, RSE serves as a quick indicator of molecular reactivity in strain-promoted reactions, suggesting a correlation between RSE and reaction outcomes rather than a causal relationship. The outcome of a reaction depends on multiple factors beyond RSE.

## Supplementary Material





## Data Availability

The AIMNet2 RSE workflow and the RSE Atlas are available at https://github.com/isayevlab/RSE_Atlas.

## Notes

More benchmarks regarding the AIMNET2 RSE Workflow; list of molecules in the copper-free click chemistry and ring opening metathesis polymerization; additional statistical distribution of RSE Atlas properties; experimental setup and 1H NMR spectra; RSE for cage-like systems (PDF)
^1^H-NMR-data (ZIP)
