# Supplementary material for: Fast and Accurate Ring Strain Energy Predictions with Machine Learning and Application in Strain-Promoted Reactions
Source: JACS Au. 2025 Oct 13;5(10):4750–61. doi: 10.1021/jacsau.5c00667 (PMC12569667; doi:10.1021/jacsau.5c00667)
Supplement: Supplementary file 1 [file au5c00667_si_001.pdf]

Supporting Information

Fast and Accurate Ring Strain Energy Predictions with Machine Learning and Application in Strain-Promoted Reactions

Zhen Liu[1#], Jessica Vinskus[1#], Yue Fu[2#], Peng Liu[3], Kevin J.T. Noonan[1], Olexandr Isayev[1]*

[#] Equal contribution

[1]Department of Chemistry, Carnegie Mellon University, Pittsburgh, PA 15213, USA

[2]Modeling and Informatics, Merck & Co., Inc., Rahway, NJ 07065, USA

[3]Department of Chemistry, University of Pittsburgh, Pittsburgh, PA, 15260, USA

[*] Correspondence: olexandr@olexandrisayev.com (O.I.)

RSE difference for different scenarios

For each scenario, we randomly selected 100 molecules. For each molecule, we selected 2 bonds that satisfied the scenario. Each bond was broken to construct the alchemical equation for RSE calculation. For the same ring, the RSEs computed using 2 different alchemical equations are called a pair of RSEs. For certain molecules, no valid 3D structure was obtained, or the thermodynamic calculation failed.

In scenario 1 (ideal breaking), 83 pairs of RSEs were obtained, with a mean absolute difference (MAD) of 0.38 kcal/mol. In scenario 2 (relaxed breaking), 75 pairs of RSEs were obtained, with a MAD of 0.37 kcal/mol. In scenario 3 (forced breaking), 64 pairs of RSEs were obtained, with a MAD of 1.55 kcal/mol. The RSE pair differences are plotted below.

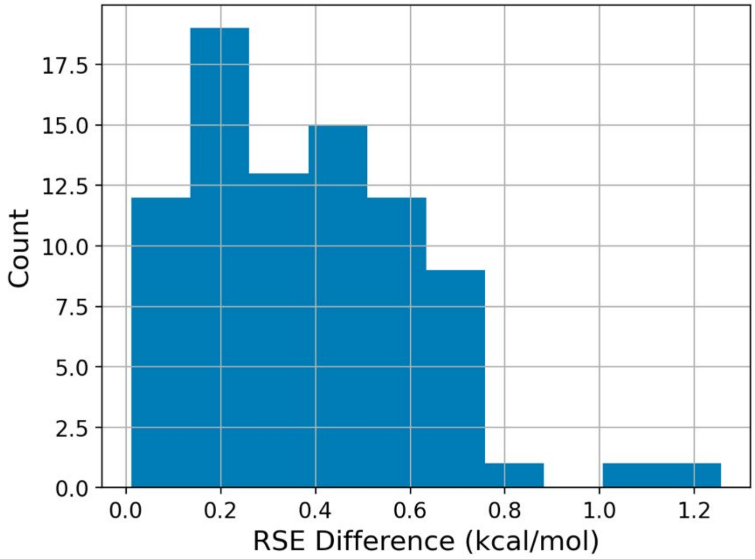

Figure S1. RSE difference in scenario 1 (ideal breaking).

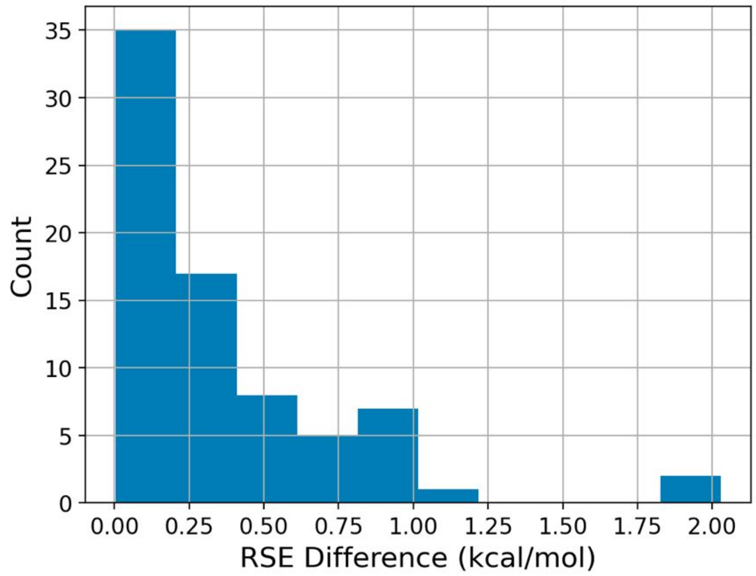

Figure S2. RSE difference in scenario 2 (relaxed breaking).

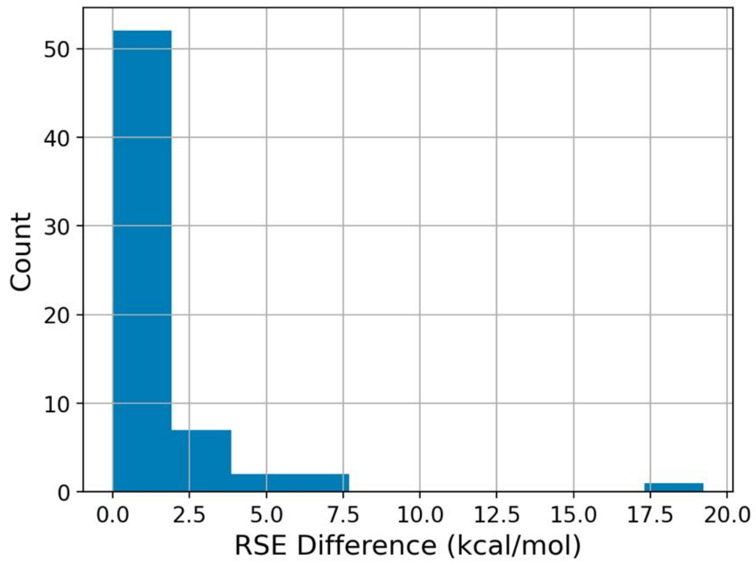

Figure S3. RSE difference in scenario 3 (forced breaking).

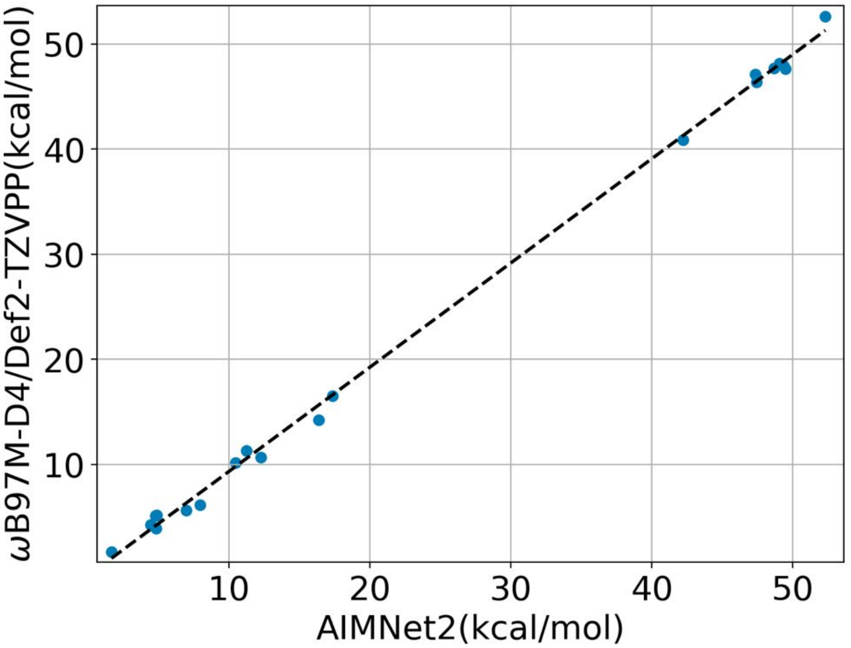

Figure S4. Correlation between the RSE calculated using the workflow and the corresponding DFT method in the validation set. The DFT method was ωB97M-D4/Def2-TZVPP in ORCA 5.0.4. The MAE and R^2 are 0.896 kcal/mol and 0.997, respectively.

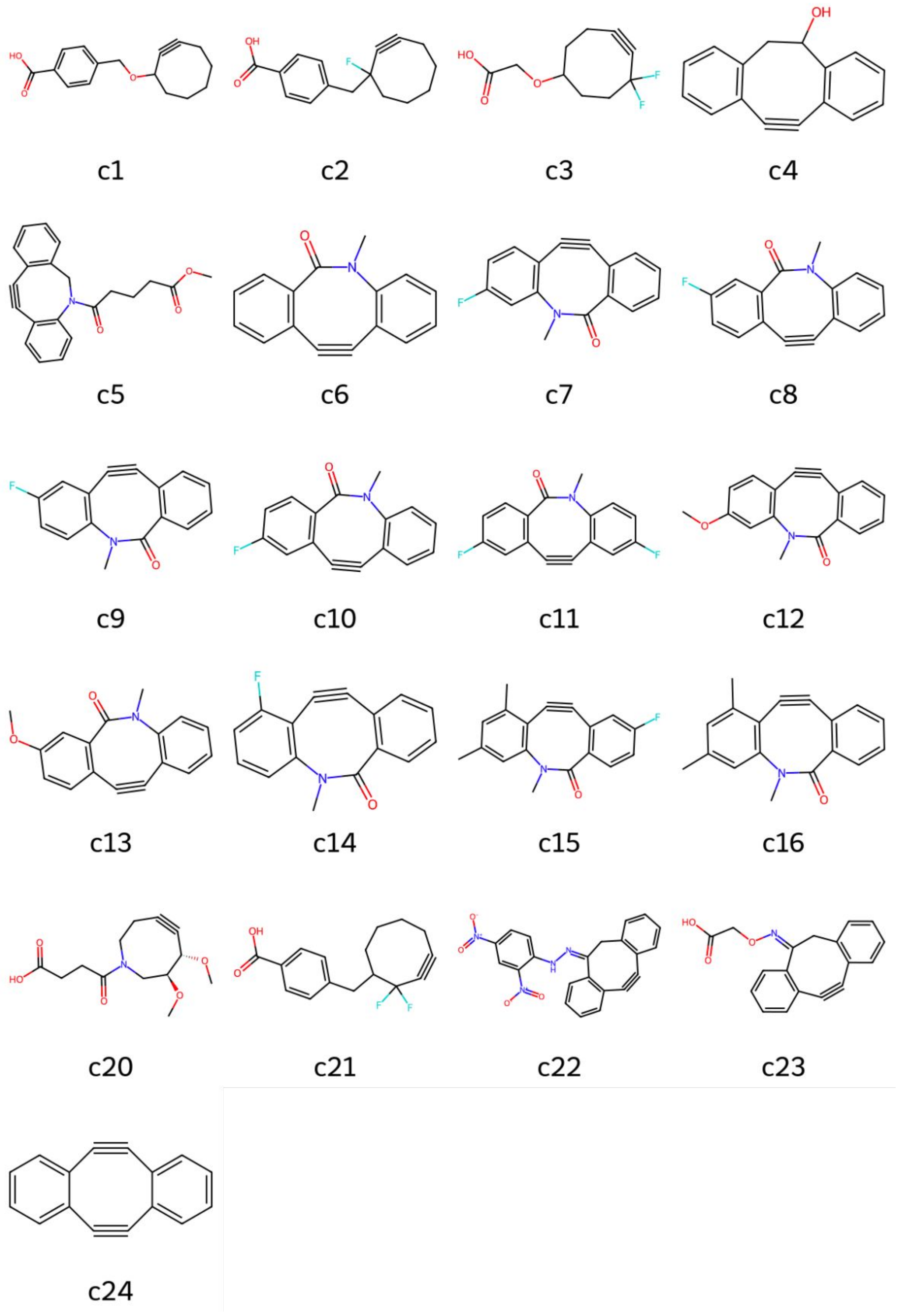

Figure S5. The list of cyclooctyne derivatives for copper-free click reactions. The molecular name is consistent with Bertozzi et al[1]. We added a character “c” preceding each number to distinguish them from other reactions investigated in this study.

Table S1. The RSE and reaction rates for the selected cyclooctyne derivatives

ID	SMILES	rate ($M^{-1}s^{-1}$)	RSE (kcal/mol)
c1	O=C(C(C=C1)=CC=C1COC2CCCCCC#C2)O	0.0024	12.57
c2	FC1(CC2=CC=C(C(O)=O)C=C2)CCCCCC#C1	0.0043	12.27
c3	FC1(F)CCC(OCC(O)=O)CCC#C1	0.077	17.32
c4	OC1C2=C(C=CC=C2)C#CC3=C(C=CC=C3)C1	0.057	11.41
c5	O=C(CCCC(OC)=O)N1C2=C(C=CC=C2)C#CC3=C (C=CC=C3)C1	0.31	13.77
c6	CN1C2=C(C=CC=C2)C#CC3=C(C=CC=C3)C1=O	0.9	25.29
c7	CN1C2=C(C=CC(F)=C2)C#CC3=C(C=CC=C3)C1= O	1.1	25.14
c8	CN1C2=C(C=CC=C2)C#CC3=C(C=C(F)C=C3)C1= O	1.1	25.72
c9	CN1C2=C(C=C(F)C=C2)C#CC3=C(C=CC=C3)C1= O	1	25.17
c10	CN1C2=C(C=CC=C2)C#CC3=C(C=CC(F)=C3)C1= O	1.2	26.36
c11	CN1C2=C(C=C(F)C=C2)C#CC3=C(C=CC(F)=C3)C 1=O	1.6	25.21
c12	CN1C2=C(C=CC(OC)=C2)C#CC3=C(C=CC=C3)C 1=O	1	25.46
c13	CN1C2=C(C=CC=C2)C#CC3=C(C=C(OC)C=C3)C 1=O	1.1	25.36
c14	CN1C2=C(C(F)=CC=C2)C#CC3=C(C=CC=C3)C1= O	0.058	26.25
c15	CN1C2=C(C(C)=CC(C)=C2)C#CC3=C(C=CC(F)=C 3)C1=O	0.0019	24.86
c16	CN1C2=C(C(C)=CC(C)=C2)C#CC3=C(C=CC=C3) C1=O	0.0009	24.84
c20	CO[C@@H]1[C@@H](OC)CN(C(CCC(O)=O)=O) CCC#C1	0.003	14.13
c21	FC1(F)C(CC2=CC=C(C(O)=O)C=C2)CCCCC#C1	0.042	15.08
c22	O=[N+](C(C=C1)=CC([N+]([O-])=O)=C1N/N=C2C C3=C(C=CC=C3)C#CC4=C/2C=CC=C4)[O-]	N/A	12.63
c23	OC(CO/N=C1CC2=C(C=CC=C2)C#CC3=C/1C=CC =C3)=O	0.061	10.98
c24	C1(C=CC=C2)=C2C#CC(C=CC=C3)=C3C#C1	0.063	4.73

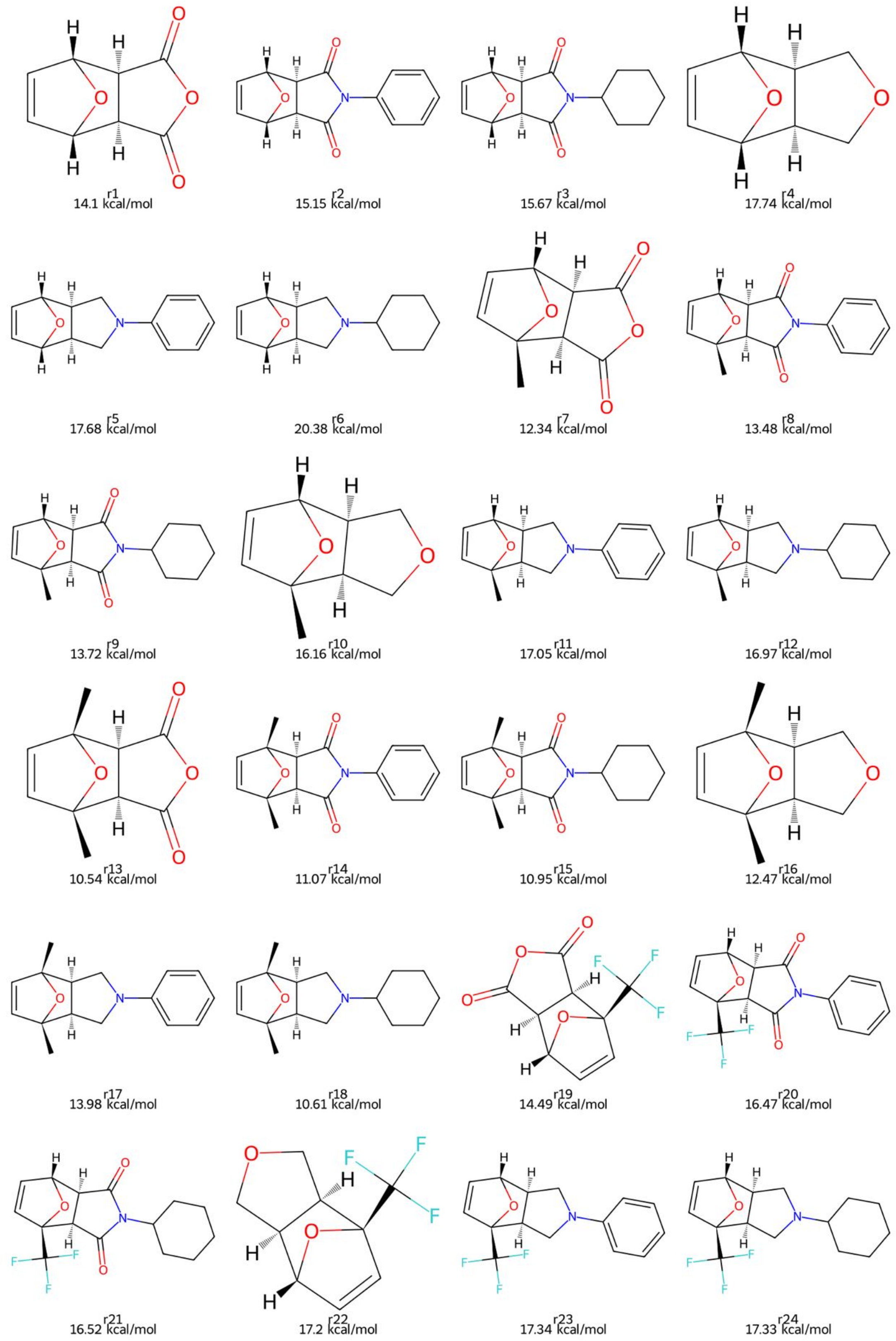

Figure S6. The list of monomers for ring opening metathesis polymerization.

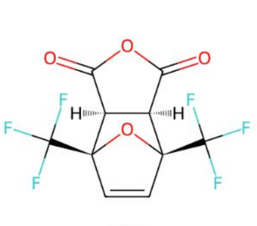

r25
12.96 kcal/mol

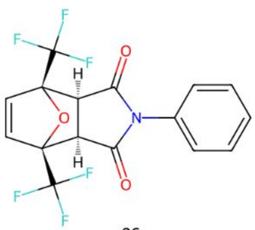

r26
12.89 kcal/mol

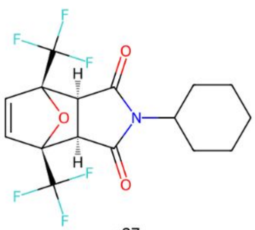

r27
14.08 kcal/mol

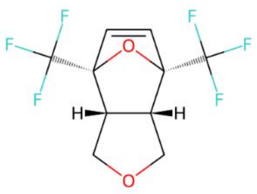

r28
12.86 kcal/mol

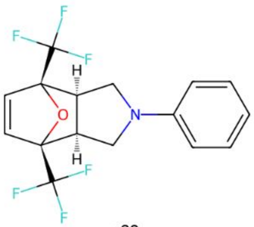

r29
13.54 kcal/mol

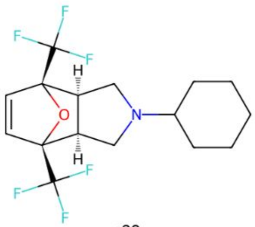

r30
11.76 kcal/mol

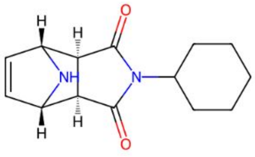

r31
16.06 kcal/mol

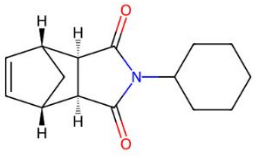

r32
15.21 kcal/mol

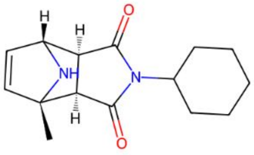

r33
17.4 kcal/mol

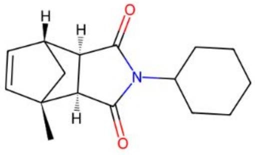

r34
15.23 kcal/mol

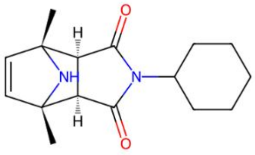

r35
10.59 kcal/mol

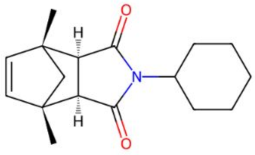

r36
10.14 kcal/mol

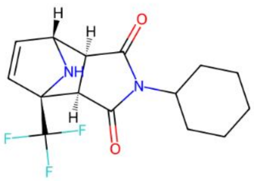

r37
18.28 kcal/mol

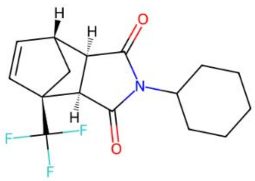

r38
14.58 kcal/mol

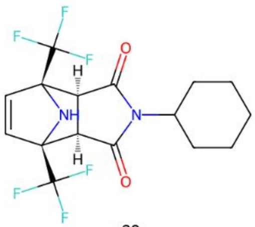

r39
11.15 kcal/mol

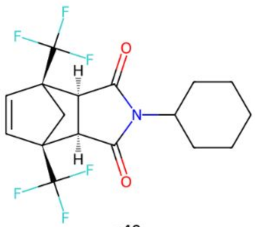

r40
8.72 kcal/mol

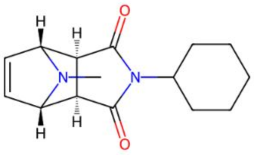

r41
17.91 kcal/mol

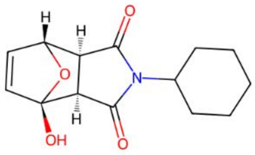

r42
17.69 kcal/mol

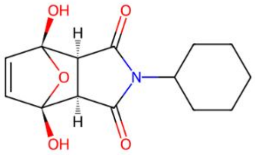

r43
18.45 kcal/mol

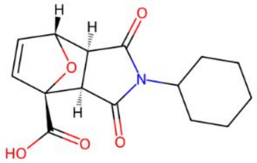

r44
18.36 kcal/mol

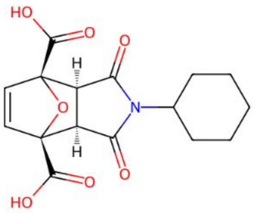

r45
12.29 kcal/mol

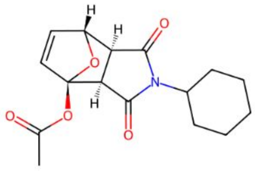

r46
18.51 kcal/mol

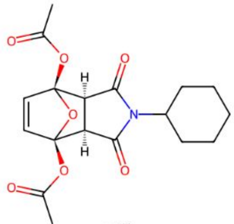

r47
16.59 kcal/mol

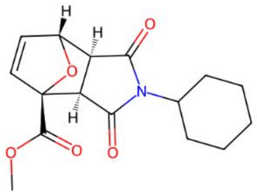

r48
17.9 kcal/mol

Figure S6. continued

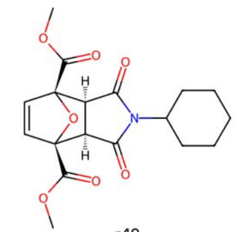

r49
14.68 kcal/mol

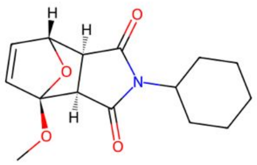

r50
20.87 kcal/mol

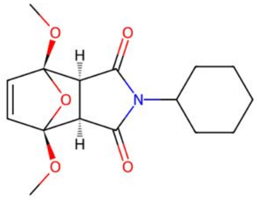

r51
21.05 kcal/mol

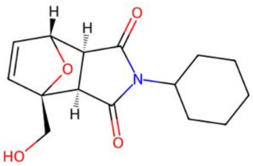

r52
14.35 kcal/mol

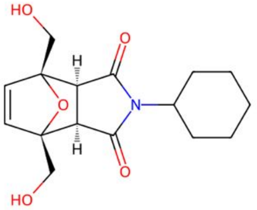

r53
12.07 kcal/mol

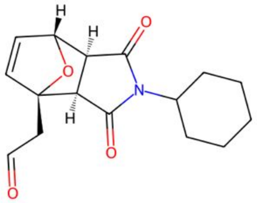

r54
19.81 kcal/mol

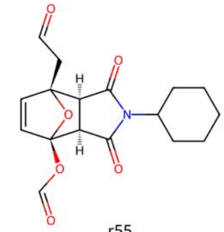

r55
14.94 kcal/mol

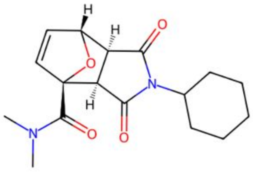

r56
16.3 kcal/mol

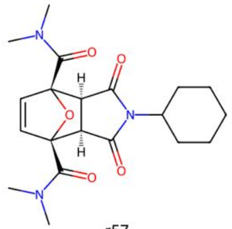

r57
13.97 kcal/mol

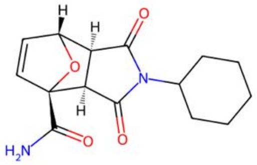

r58
17.61 kcal/mol

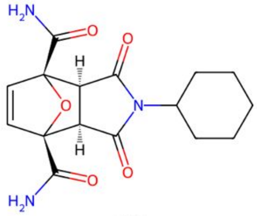

r59
17.05 kcal/mol

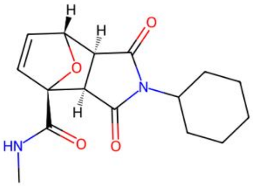

r60
18.04 kcal/mol

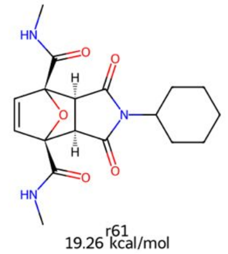

r61
19.26 kcal/mol

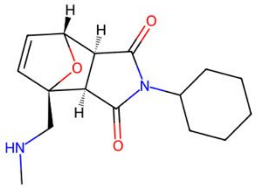

r62
15.96 kcal/mol

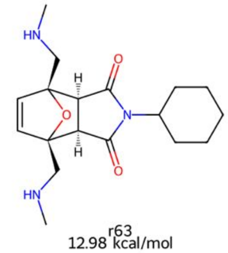

r63
12.98 kcal/mol

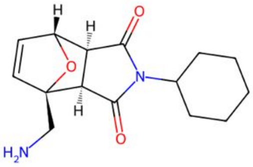

r64
14.81 kcal/mol

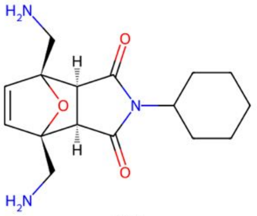

r65
15.08 kcal/mol

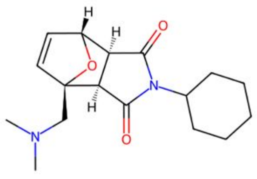

r66
16.44 kcal/mol

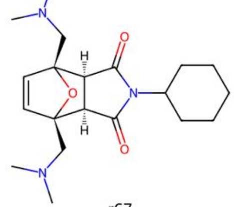

r67
14.33 kcal/mol

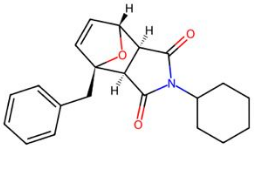

r68
14.36 kcal/mol

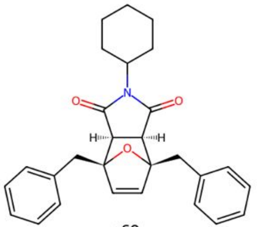

r69
10.44 kcal/mol

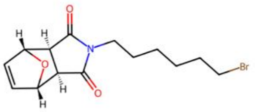

r70
13.58 kcal/mol

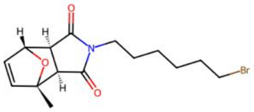

r71
11.39 kcal/mol

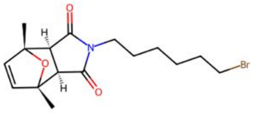

r72
10.2 kcal/mol

Figure S6. continued

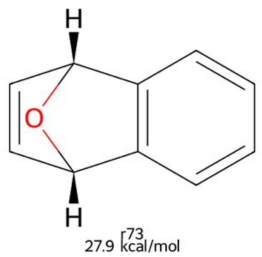
r73 27.9 kcal/mol

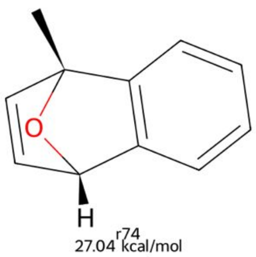
r74 27.04 kcal/mol

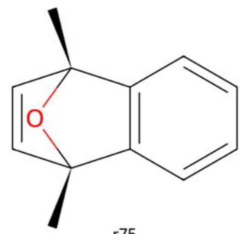
r75 25.14 kcal/mol

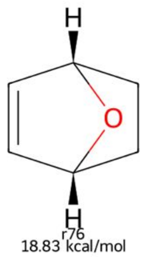
r76 18.83 kcal/mol

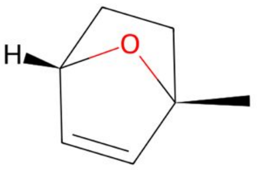
r77 17.83 kcal/mol

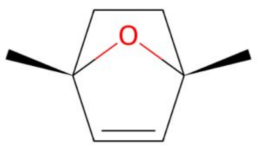
r78 16.19 kcal/mol

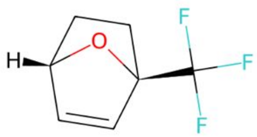
r79 19.74 kcal/mol

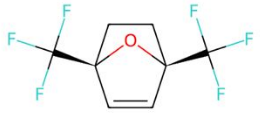
r80 19.38 kcal/mol

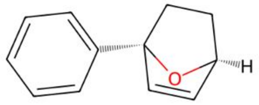
r81 18.83 kcal/mol

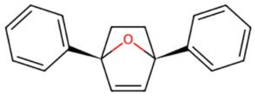
r82 17.66 kcal/mol

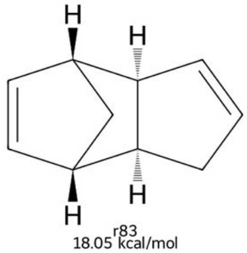
r83 18.05 kcal/mol

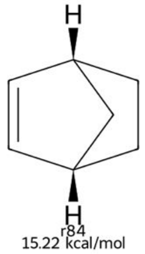
r84 15.22 kcal/mol

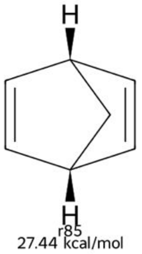
r85 27.44 kcal/mol

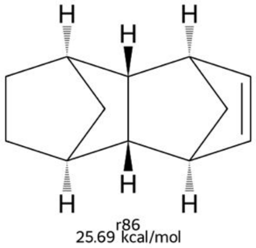
r86 25.69 kcal/mol

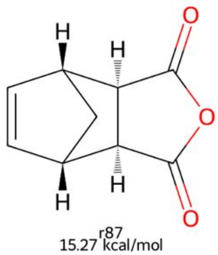
r87 15.27 kcal/mol

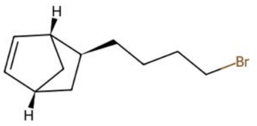
r88 18.37 kcal/mol

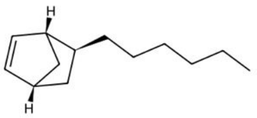
r89 18.86 kcal/mol

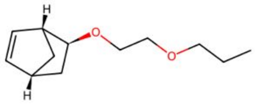
r90 17.85 kcal/mol

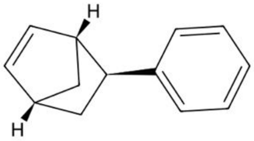
r91 19.09 kcal/mol

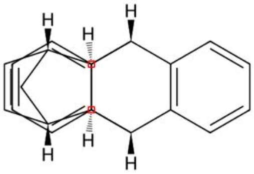
r93 22.68 kcal/mol

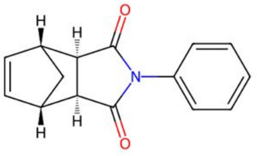
r94 15.17 kcal/mol

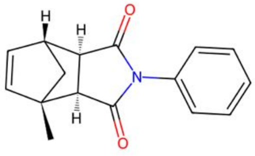
r95 15.21 kcal/mol

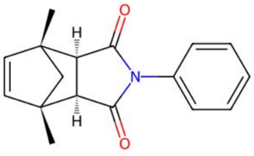
r96 9.96 kcal/mol

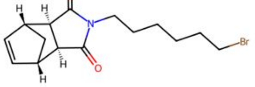
r97 17.5 kcal/mol

Figure S6. continued

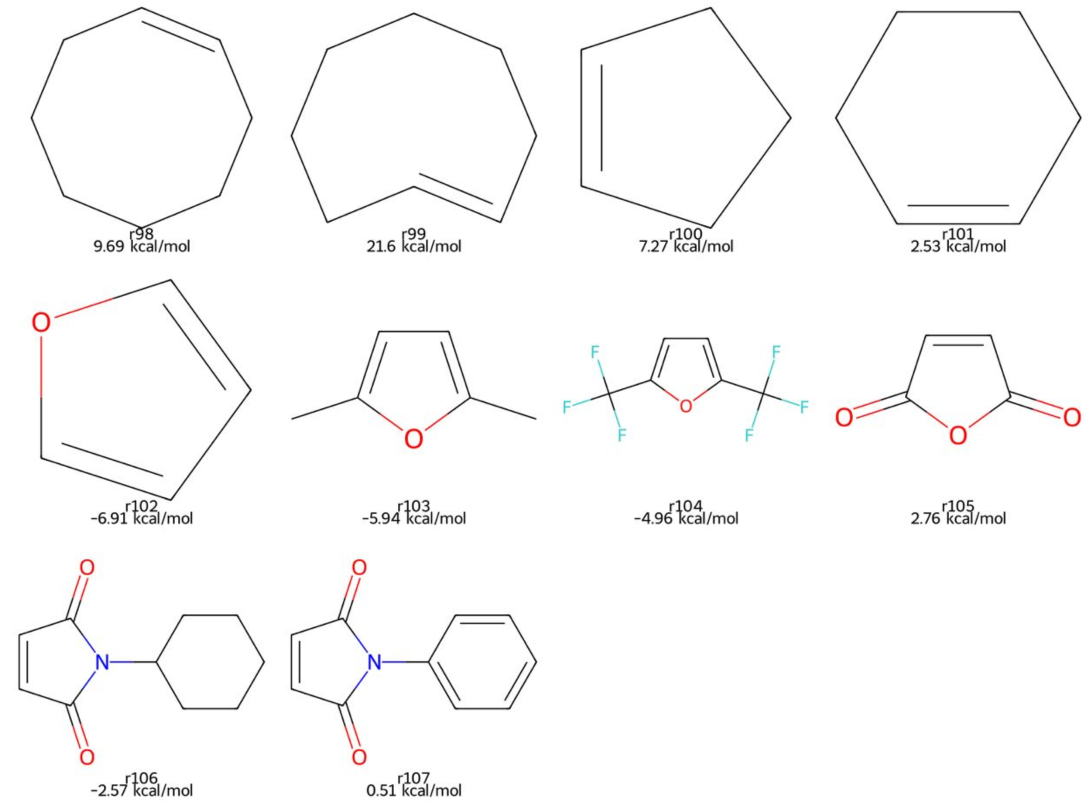

Figure S6. continued

Ring Atlas validation

For the computational Ring Atlas, we sampled 130 molecules for DFT verification. The sampling strategy is as follows: we sampled the top and bottom rings as ranked by the ring strain energy for each group of molecules. A group of molecules is the set of all molecules with the same ring size. There are 7 groups, where the ring size ranges from 3 to 9. For DFT verification, we conducted geometry optimization and frequency analysis using the conformers in our ring atlas dataset as the starting point. Some DFT jobs failed either due to convergence issue or very rare structure. We ended up with 77 pairs of successful DFT calculation results. The correlation between the RSE computed using the DFT method and the workflow is show below. The R^2, MAE, RMSE are 0.99, 1.76 kcal/mol and 2.41 kcal/mol, respectively.

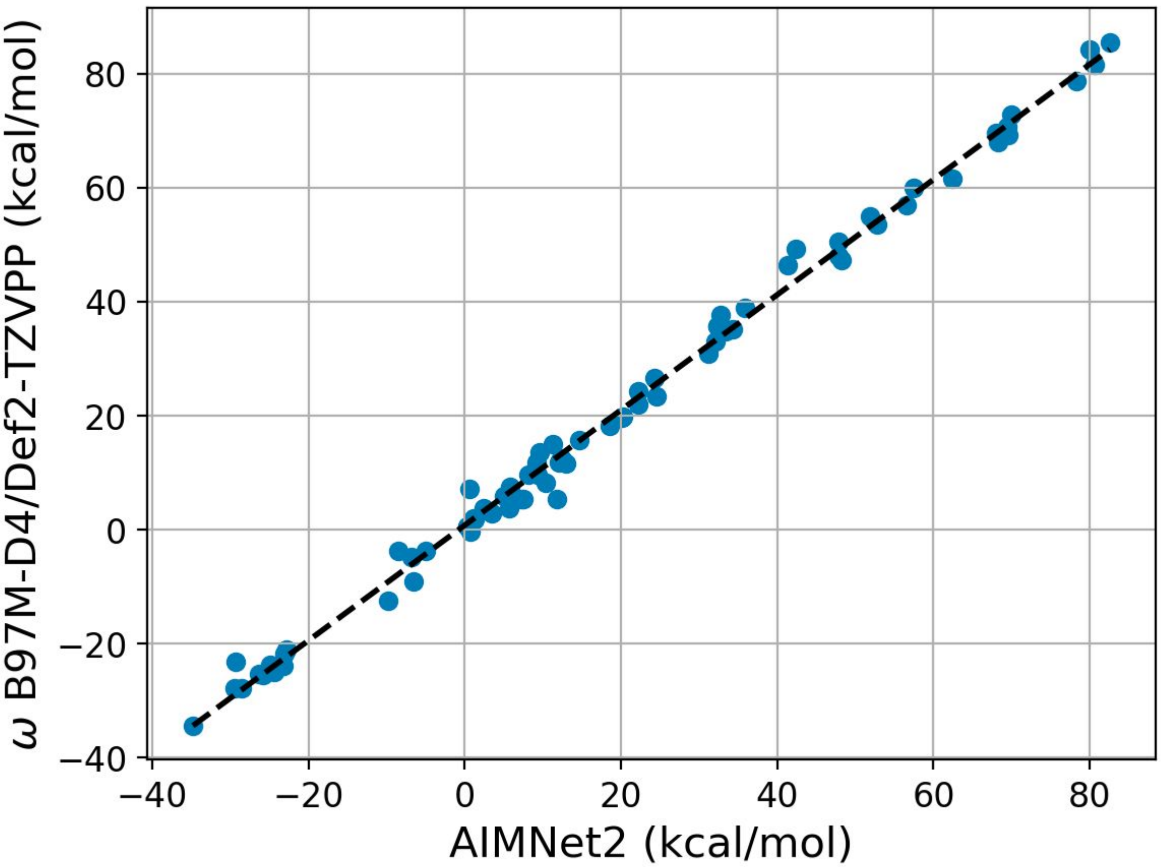

Figure S7. RSE correlation computed from the DFT method and the AIMNet2 model in the workflow.

Characteristics of the Ring Atlas

The following figures describes the characteristics of the ring atlas. **Figure S8** and **Figure S9** shows the distribution of ring size and RSE, respectively. **Figure S10** shows how the RSE distribution changes as the ring size changes, and **Figure S11** shows how the RSE distribution changes as the ring unsaturation changes. For each ring, the total unsaturation is defined as followings:

$$\text{Total unsaturation} = \text{number of double bonds} + 2 \times \text{number of triple bonds}$$

For **Figure S11**, the RSE distributions for rings with total unsaturation greater than 6 are not shown due to small numbers.

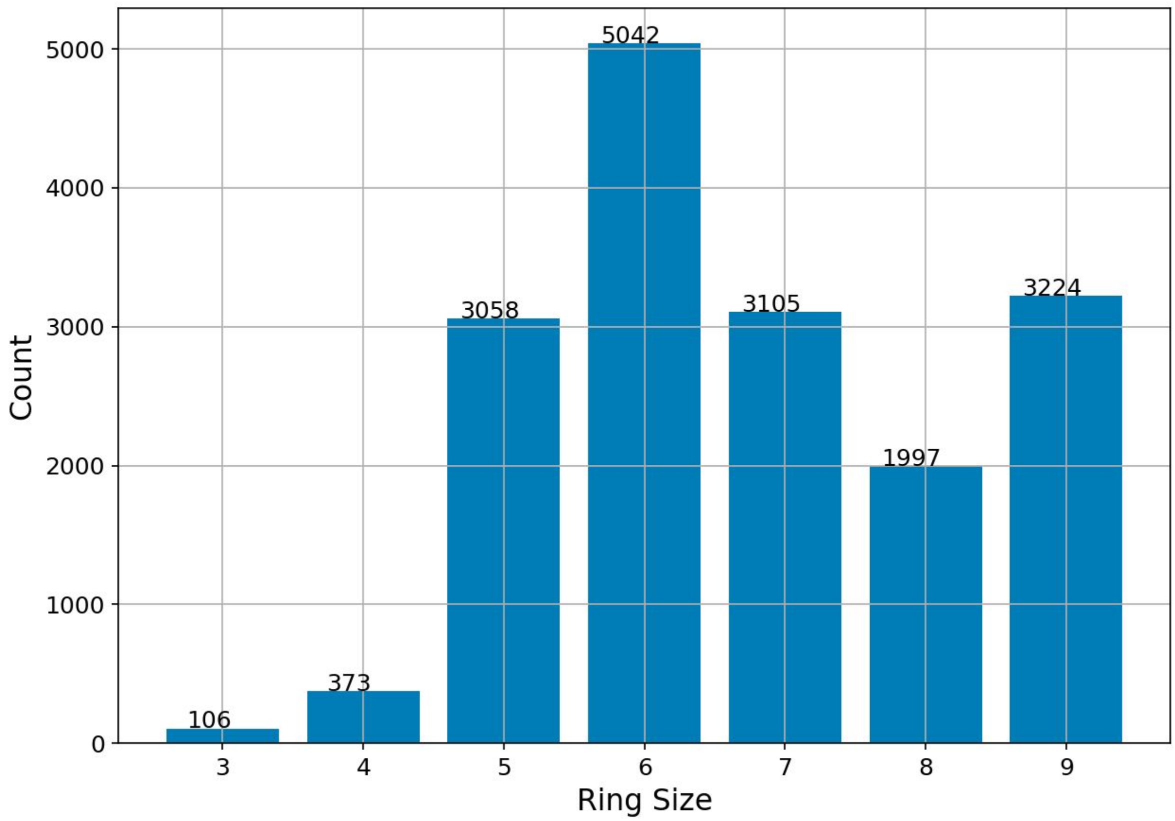

Figure S8. The number of rings with each size.

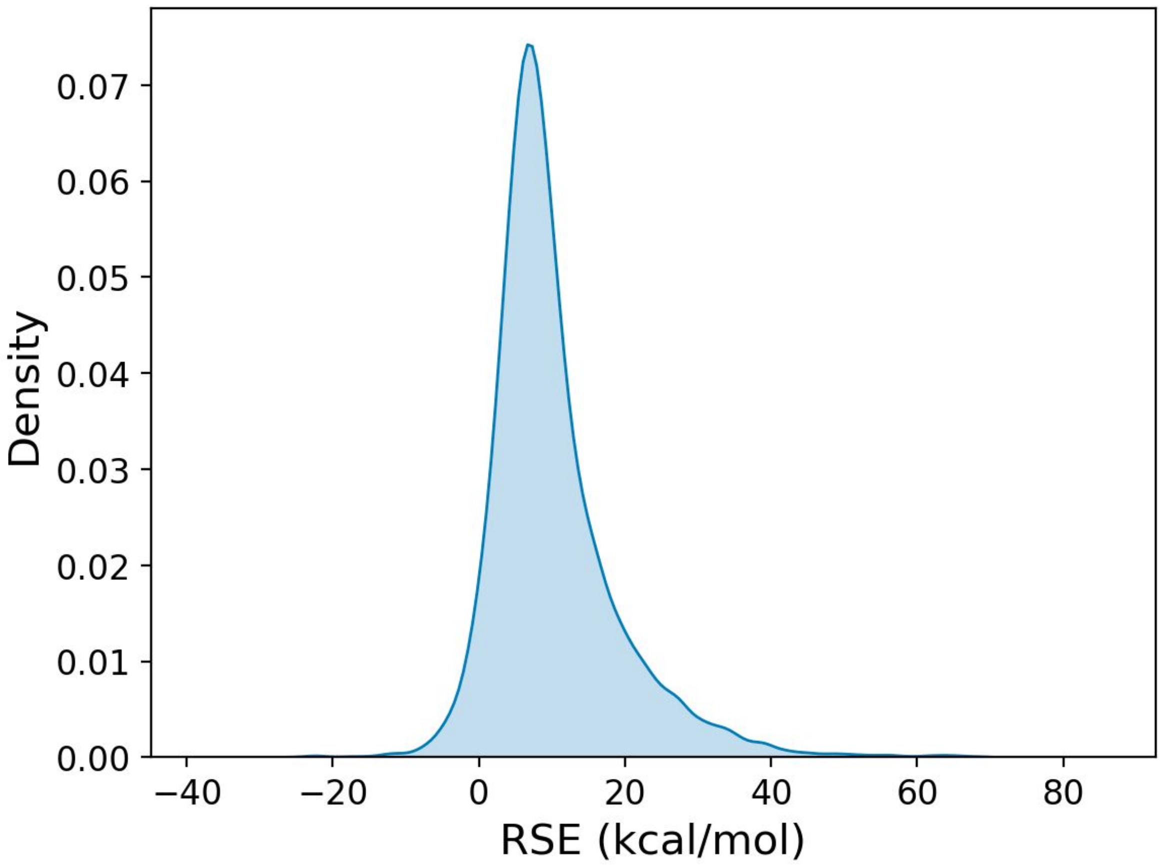

Figure S9. The RSE distribution of the Ring Atlas.

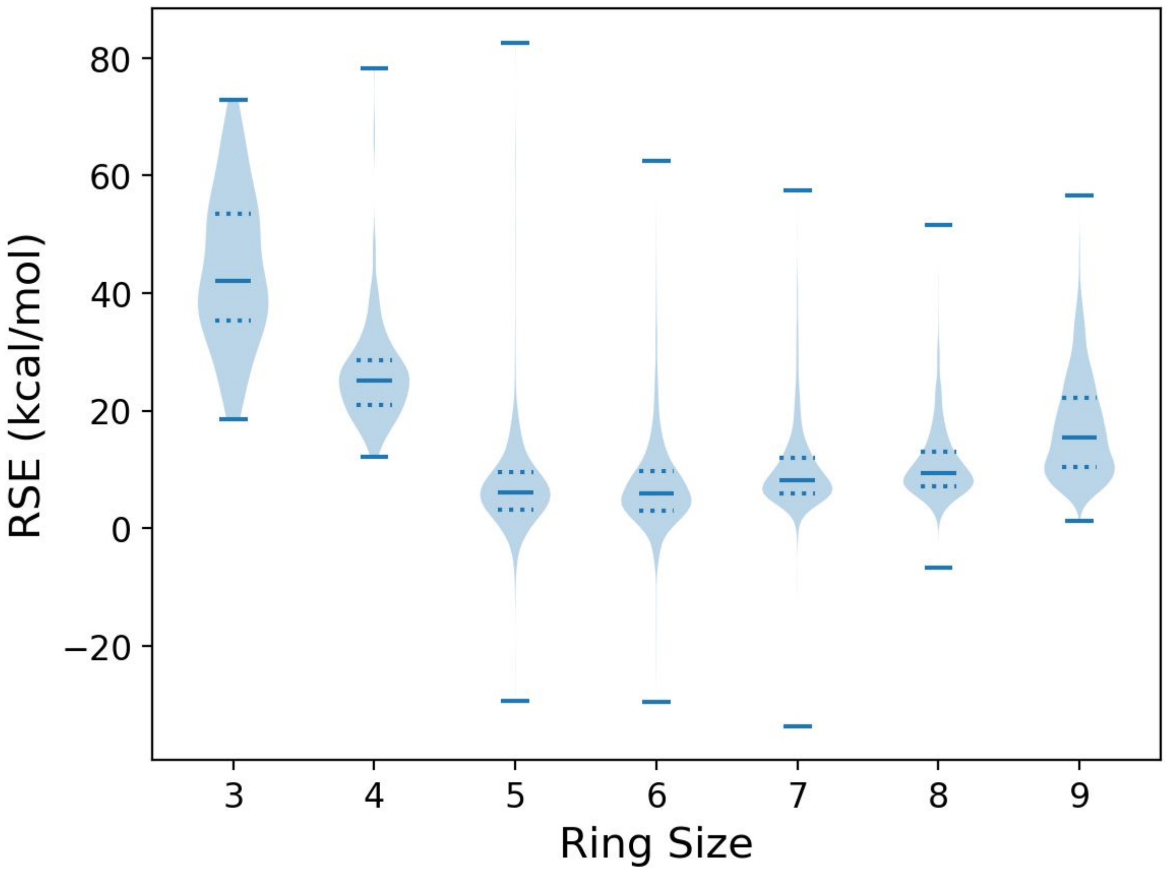

Figure S10. The RSE energy distribution for different ring size groups. For each group, horizontal lines from top to bottom represent the maximum, 75 percentile, median, 25 percentile and minimum RSE in this group. The width of the violin group represents the density of molecules with that RSE.

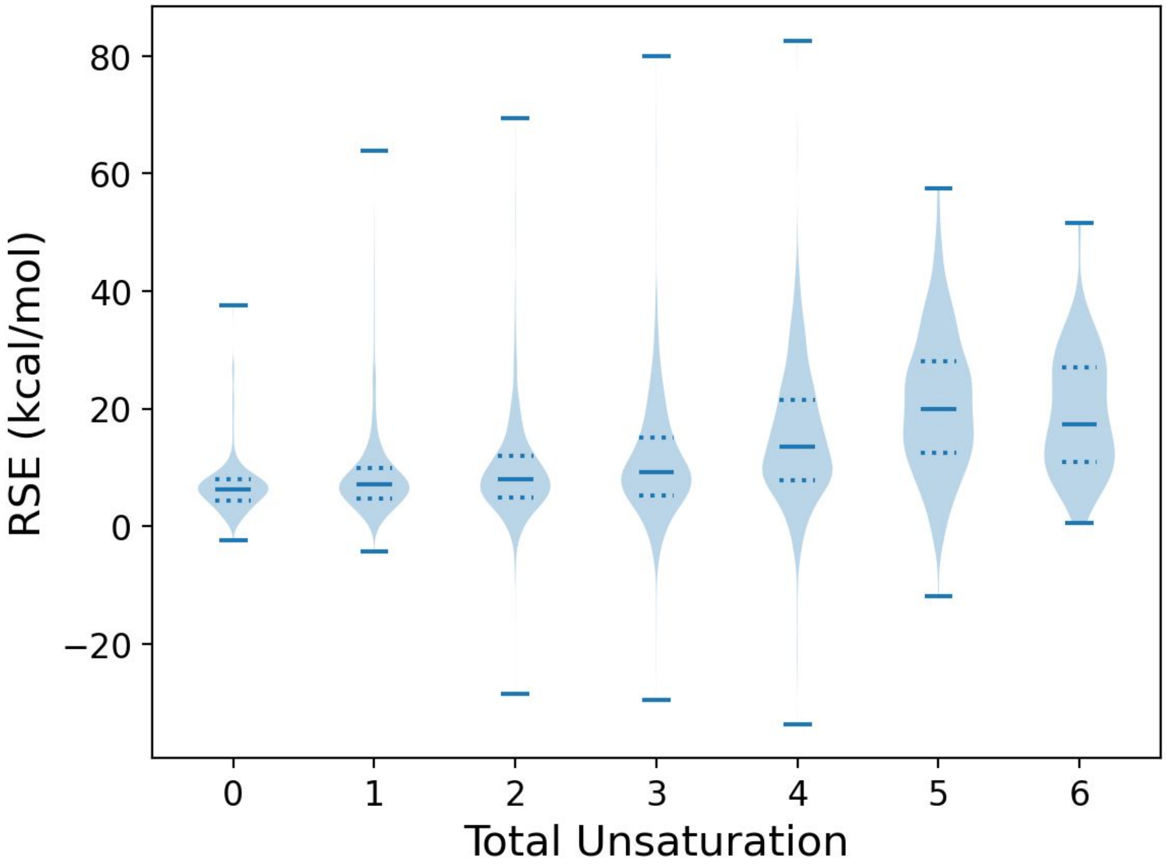

Figure S11. The RSE distribution for each unsaturation group.

Experimental Methods

Materials

Norbornadiene, Norbornene, Cyclohexene, and Cyclooctene were obtained from commercial vendors and used without further purification. Hexylnorbornene[2], Bromobutylnorbornene[3], 1,4-dimethyl-1,4-dihydro-1,4-epoxynapthalene[4], and Norbornene-anthracene cycloadduct[3] were synthesized by previously reported procedures. Nuclear magnetic resonance spectroscopy (NMR) was performed on a 500 MHz Bruker Avance 3 Spectrometer or a 500 MHz Bruker Neo Spectrometer with Prodigy Cryoprobe. The ^{1}H NMR spectra were referenced to residual protio solvents (7.26 ppm for $CHCl_3$).

General Synthesis of 7-Oxanorbornene Derivatives

These compounds were synthesized in a similar fashion to previous reports and ^{1}H NMR data is included for reference below.[5] Anywhere from 1.5-3 equiv. of a furan derivative was

dissolved in ethyl acetate in a round bottom flask equipped with a stir bar. To the rapidly stirring furan solution, 1 equiv. of a maleimide was added. The reaction flask was then equipped with a condenser and heated to 60 °C for 5 h. The vessel was then cooled to room temperature. Solvent was removed via rotary evaporation, and the solids were redissolved in ethyl acetate, and precipitated into hexanes. The resultant powder was then recrystallized, by dissolving the product in ethyl acetate and adding hexanes until the mixture was slightly cloudy. This solution was then heated until everything fully dissolved and allowed to recrystallize slowly for several hours upon cooling to room temperature. The resultant solid was then isolated by vacuum filtration to yield the desired oxanorbornene.

***N*-cyclohexyl-7-oxanorbornenedicarboximide (2-cyclohexyl-3a,4,7,7a-tetrahydro-1H-4,7-epoxyisoindole-1,3(2H)-dione) - CyONDI**

5.00 g (28 mmol) of *N*-cyclohexylmaleimide, 6.2 mL (85 mmol) of furan, 100 mL of ethyl acetate. The product, a mixture of *endo* and *exo* isomers (47:53), was isolated as a white powder (1.86 g, 27% yield). *exo-N*-cyclohexyl-7-oxanorbornenedicarboximide ^{1}H NMR (500 MHz, $CDCl_3$) δ 6.49, (t, J_{HH} = 0.9 Hz , 2H), δ 5.24, (t, J_{HH} = 0.5 Hz, 2H), δ 3.91, (tt, J_{HH} = 12.4, 3.8 Hz, 1H), δ 2.75, (s, 2H), δ 2.11, (qd, J_{HH} = 12.5, 3.1 Hz, 2H), δ 1.80 (m, 2H, overlaps with a peak from the other isomer), δ 1.63 (m, 3H, overlaps with a peak from the other isomer), δ 1.27 (m, 3H, overlaps with a peak from the other isomer). *endo-N*-cyclohexyl-7-oxanorbornenedicarboximide ^{1}H NMR (500 MHz, $CDCl_3$) δ 6.38, (t, J_{HH} = 1 Hz, 2H), δ 5.30, (m, 2H), δ 3.75, (tt, J_{HH} = 12.4, 3.8 Hz, 1H), δ 3.43, (dd J_{HH} = 3.6, 1.8 Hz, 2H), δ 2.00, (qd, J_{HH} = 12.5, 3.1 Hz, 2H), δ 1.80 (m, 2H, overlaps with a peak from the other isomer), δ 1.63 (m, 1H, overlaps with a peak from the other isomer), δ 1.47 (m, 2H), δ 1.27 (m, 3H, overlaps with a peak from the other isomer).

1-methyl-*N*-cyclohexyl-7-oxanorbornenedicarboximide (2-cyclohexyl-4-methyl-3a,4,7,7a-tetrahydro-1H-4,7-epoxyisoindole-1,3(2H)-dione) - MCyONDI

5.72 g (32 mmol) of *N*-cyclohexylmaleimide, 4.4 mL (49 mmol) of 2-methylfuran, 23 mL of ethyl acetate. The *exo* product was obtained as a white crystalline powder (4.99 g, 59%). ^{1}H NMR (500 MHz, $CDCl_3$) δ 6.48 (d, J_{HH} = 5.6 Hz, 1H), δ 6.29 (d, J_{HH} = 5.6 Hz, 1H), δ 5.15 (d, J_{HH} = 1.5 Hz, 1H), δ 3.91 (tt, J_{HH} = 12.3, 4.0 Hz, 1H), δ 2.87 (d, J_{HH} = 6.4 Hz, 1H), δ 2.60 (d, J_{HH} = 6.4 Hz, 1H), δ 2.12 (q, J_{HH} = 12.5 Hz, 2H), δ 1.81 (q, J_{HH} = 13.2 Hz, 2H), δ 1.70 (s, 3H), δ 1.63 (m, 3H), δ 1.25 (m, 3H).

1,4-dimethyl-*N*-cyclohexyl-7-oxanorbornenedicarboximide (2-cyclohexyl-4,7-dimethyl-3a,4,7,7a-tetrahydro-1H-4,7-epoxyisoindole-1,3(2H)-dione) - (DMCyONDI)

5.54 g (31 mmol) of *N*-cyclohexylmaleimide, 7.5 mL (70 mmol) of 2,5-dimethylfuran, 100 mL of ethyl acetate. The *exo* product was obtained (3.53 g (41%)) as white needle-like crystals. ^{1}H NMR (500 MHz, $CDCl_3$) δ 6.28 (s, 2H), δ 3.92 (tt, J_{HH} = 12.5, 3.7, 1H), δ 2.72 (s, 2H), δ 2.13 (qd, J_{HH} = 12.5, 3.4 Hz, 2H), δ 1.81 (d, J_{HH} = 13.4 Hz, 2H), δ 1.62 (m, 10H), δ 1.26 (m, 4H).

***N*-phenyl-7-oxanorbornenedicarboximide (2-phenyl-3a,4,7,7a-tetrahydro-1H-4,7-epoxyisoindole-1,3(2H)-dione) - PhONDI**

1g (5.8 mmol) of *N*-Phenylmaleimide, 1.2 mL (17 mmol) of furan, 20 mL of ethyl acetate. The product, a mixture of *endo and exo* isomers (40:60), was isolated as a white powder (0.73 g, 52% yield). *endo*-N-phenyl-7-oxanorbornenedicarboximide ^{1}H NMR (500 MHz, $CDCl_3$) δ 7.44 (m, 3H, overlaps with a peak from the other isomer), δ 7.28 (m, 1H, overlaps with solvent residual peak and a peak from the other isomer), δ 7.11 (m, 1H, overlaps with a peak from the other isomer), δ 6.57 (t, J_{HH} = 1.0 Hz, 2H), δ 5.40 (t, J_{HH} = 1.0 Hz, 2H), δ 3.01 (s, 2H). *exo*- N-phenyl-7-oxanorbornenedicarboximide ^{1}H NMR (500 MHz, $CDCl_3$) δ 7.45 (m, 3H, overlaps with a peak from the other isomer), δ 7.30 (m, 1H, overlaps with a peak from the other isomer), δ 7.14 (m, 1H overlaps with a peak from the other isomer), δ 6.57 (t, J_{HH} = 1.0 Hz, 2H), δ 5.44 (m, 2H), δ 3.69 (dd, , J_{HH} = 3.6, 1.62 Hz, 2H).

1-methyl-*N*-phenyl-7-oxanorbornenedicarboximide (4-methyl-2-phenyl-3a,4,7,7a-tetrahydro-1H-4,7-epoxyisoindole-1,3(2H)-dione) - MPhONDI

5.64g (32 mmol) of *N*-Phenylmaleimide, 4.4 mL (48 mmol) of 2-methyl furan, 23 mL of ethyl acetate. The *exo* product was obtained as a white, crystalline powder (6.9 g, 83%). ^{1}H NMR (500 MHz, $CDCl_3$) δ 7.47 (t, 2H), δ 7.39 (tt, J_{HH} = 7.4, 1.2 Hz, 1H), δ 7.28 (m, 2H), δ 6.56 (dd, J_{HH} = 5.7, 1.7 Hz, 1H), δ 6.37 (d, J_{HH} = 5.7 Hz, 1H), δ 5.31 (d, J_{HH} = 1.8 Hz, 1H), δ 3.13 (d, J_{HH} = 6.4 Hz, 1H), δ 2.86 (d, J_{HH} = 6.5 Hz, 1H), δ 1.80 (s, 3H).

1,4-dimethyl-*N*-phenyl-7-oxanorbornenedicarboximide (4,7-dimethyl-2-phenyl-3a,4,7,7a-tetrahydro-1H-4,7-epoxyisoindole-1,3(2H)-dione) - DMPhONDI

0.98g (5.2 mmol) of *N*-Phenylmaleimide, 1.9 mL (18 mmol) of 2,5-dimethyl furan,19 mL of ethyl acetate. The *exo* product was obtained as colorless prismatic crystals (0.57 g, 38%). ^{1}H NMR (500 MHz, $CDCl_3$) δ 7.47 (t, J_{HH} = 7.7 Hz, 2H), δ 7.38 (tt, J_{HH} = 7.5, 2.0 Hz, 1H), δ 7.27 (m, 2H), δ 6.37 (s, 2H), δ 2.98 (s, 2H), δ 1.77 (s, 6H).

ROMP Monomer Consumption Experiments

In a glove box, a 50 mM stock solution of Grubbs third generation catalyst (G3) was prepared by combining 73.7 mg (0.10 mmol) of catalyst and dry dichloromethane in a 2 mL volumetric flask (solvent added to fill to the mark). Then, 0.1 mL (0.005 mmol) of the stock solution was transferred to a 20 mL vial and equipped with a stir bar, then tightly capped with a septum cap, and removed from the glove box. A 500 mM stock solution of trimethoxybenzene (TMB) was prepared by adding 424 mg (2.5 mmol) to a 5 mL volumetric flask and diluting with dry dichloromethane. Then 0.5 mL (0.25 mmol) of the TMB stock was added to 1 mmol of monomer along with 1.4 mL of dry dichloromethane. A 0.05 mL aliquot of this monomer solution was removed and diluted with 0.5 mL of $CDCl_3$ to establish the concentration at $t = 0$ using ^1H NMR spectroscopy. The vial containing the catalyst was placed into a 35 °C oil bath, while stirring at 600 rpm. The monomer solution was then quickly injected into the rapidly stirring catalyst solution and stirred for 1 h. A 0.1 mL aliquot was then removed from the reaction mixture, diluted with 0.5 mL $CDCl_3$, and analyzed by ^1H NMR spectroscopy to determine monomer consumption. If less than 90% complete, the reaction was allowed to stir for 23 h more before another aliquot was taken and analyzed for completion by NMR.

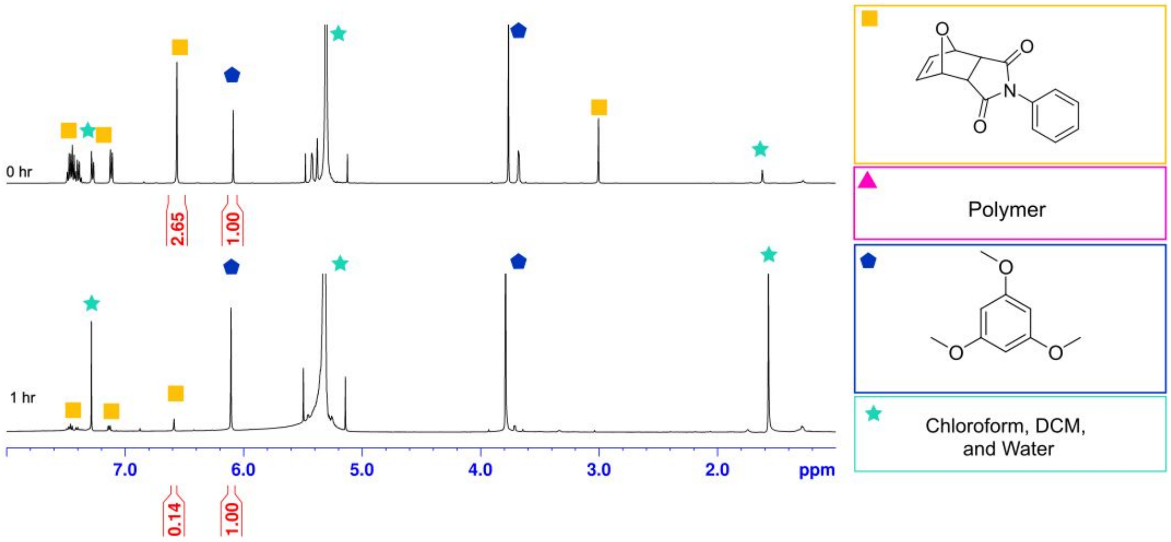

Figure S12. ^{1}H NMR data of monomer PhONDI (r2). The polymer signals are not visible due to lack of solubility.

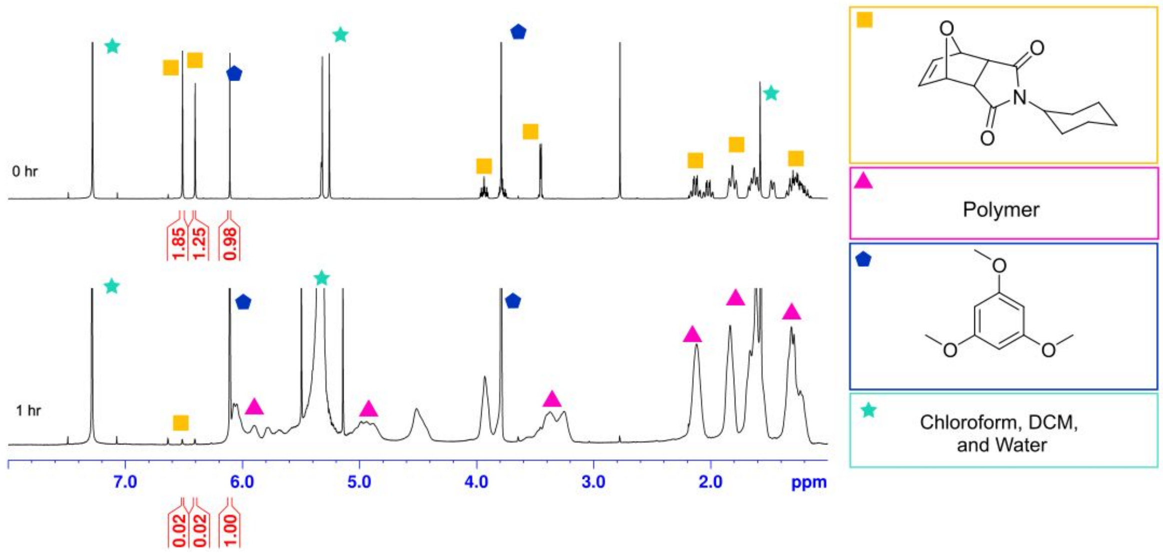

Figure S13. ^{1}H NMR data of monomer CyONDI (r3) and the corresponding polymer.

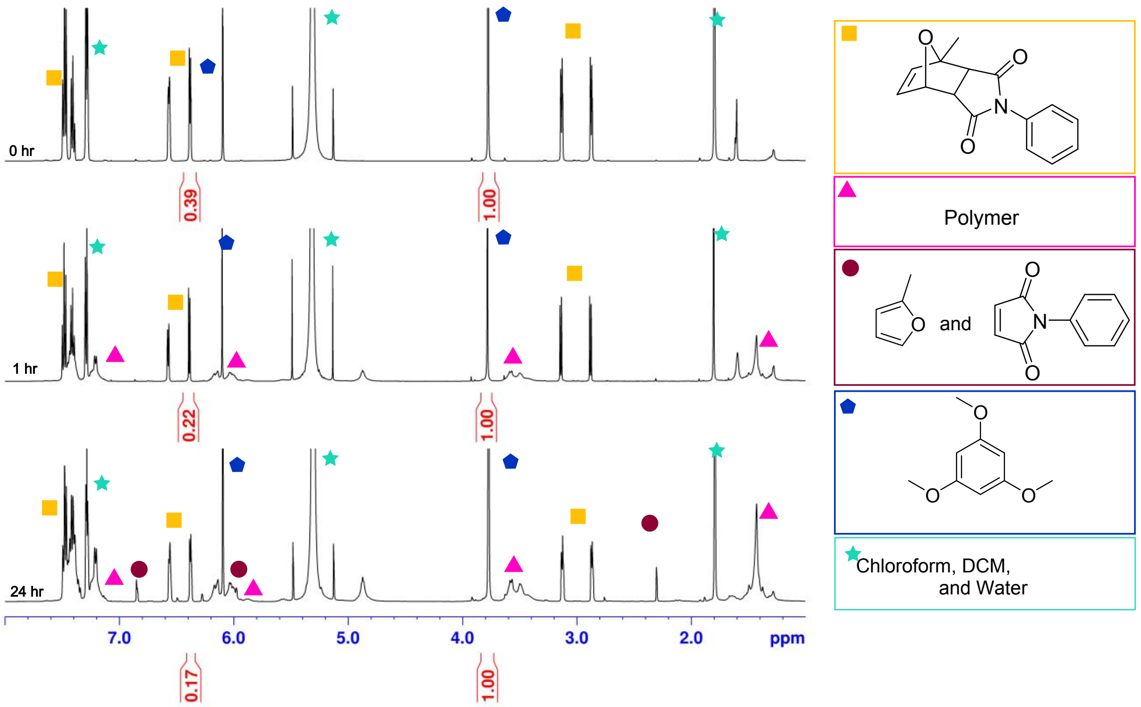

Figure S14. ^{1}H NMR data of monomer MPhONDI (r8) and the corresponding polymer.

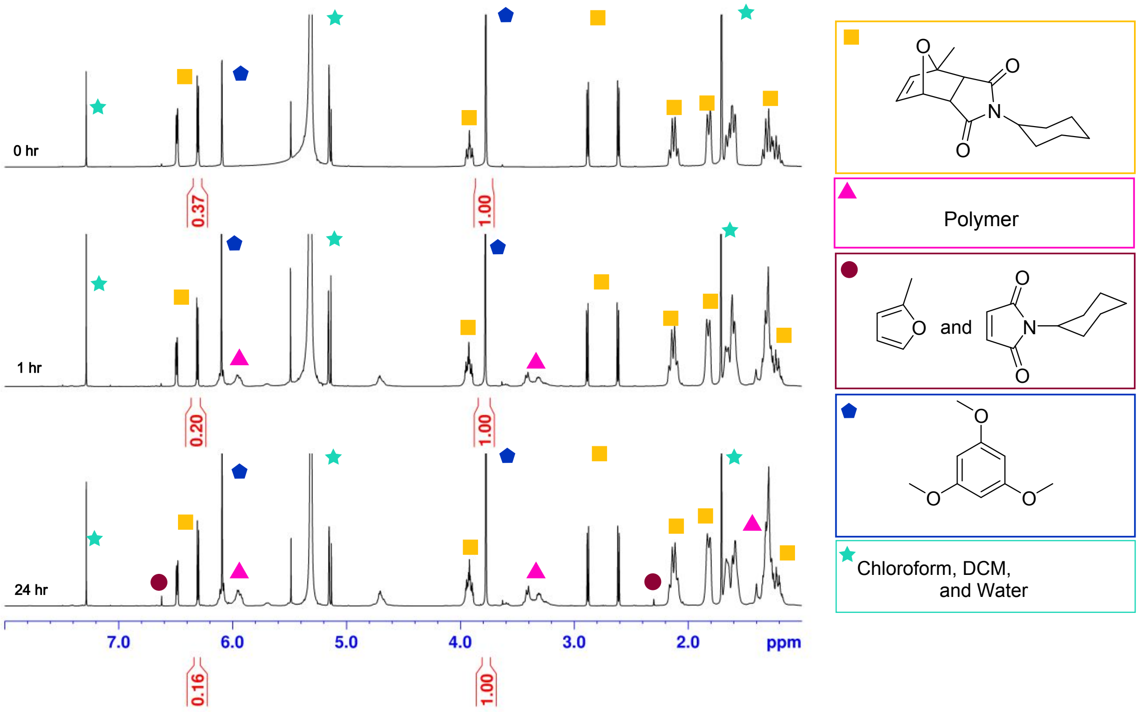

Figure S15. ^{1}H NMR data of monomer MCyONDI (r9) and the corresponding polymer.

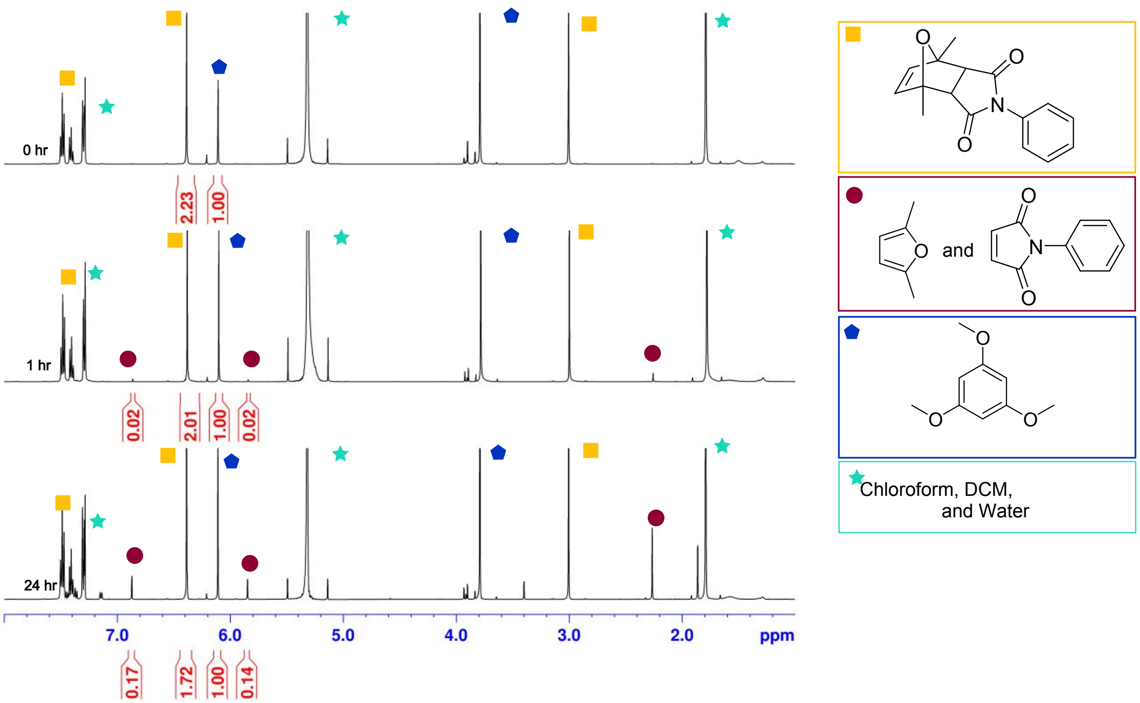

Figure S16. ^{1}H NMR of monomer DMPhONDI (r14) and the retro-Diels-Alder products.

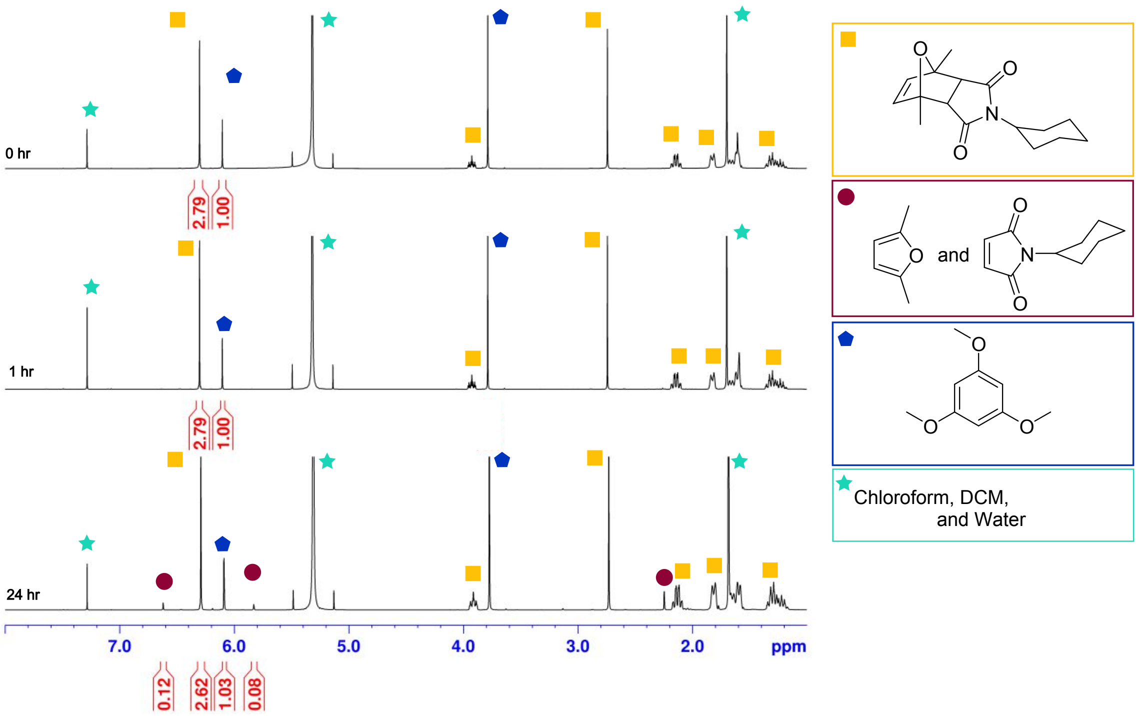

Figure S17. ^{1}H NMR data of monomer DMCyONDI (r15) and the retro-Diels-Alder products.

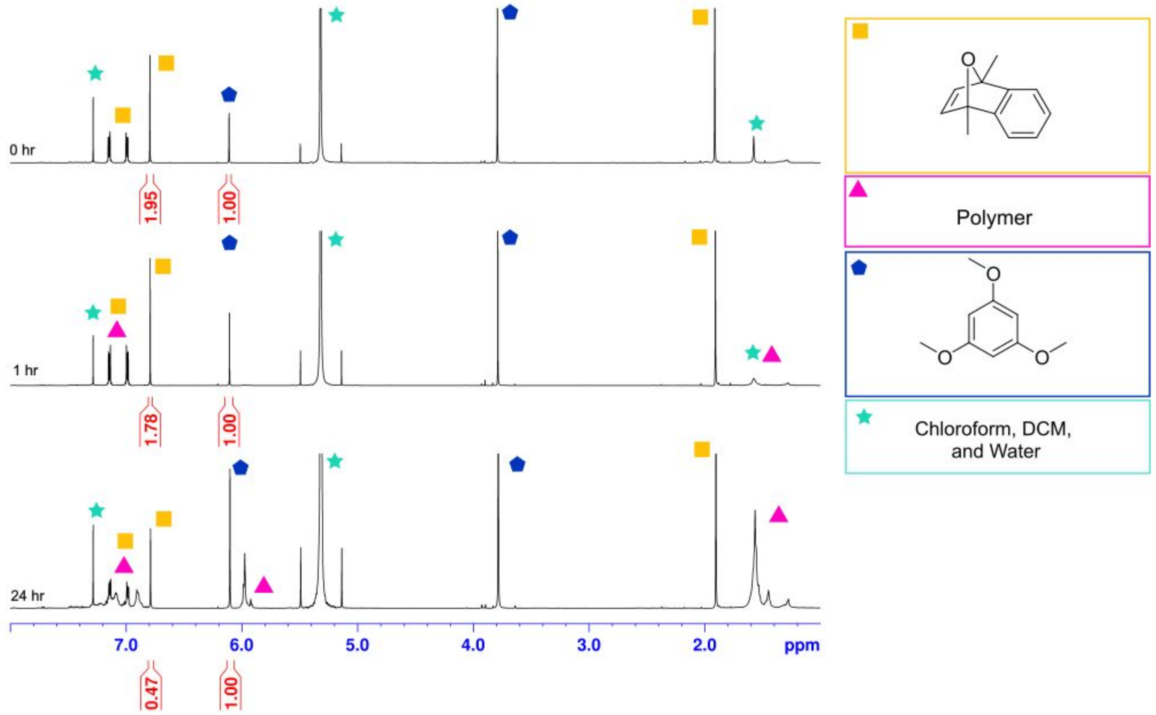

Figure S18. ^{1}H NMR data of monomer DMONa (r75) and the corresponding polymer.

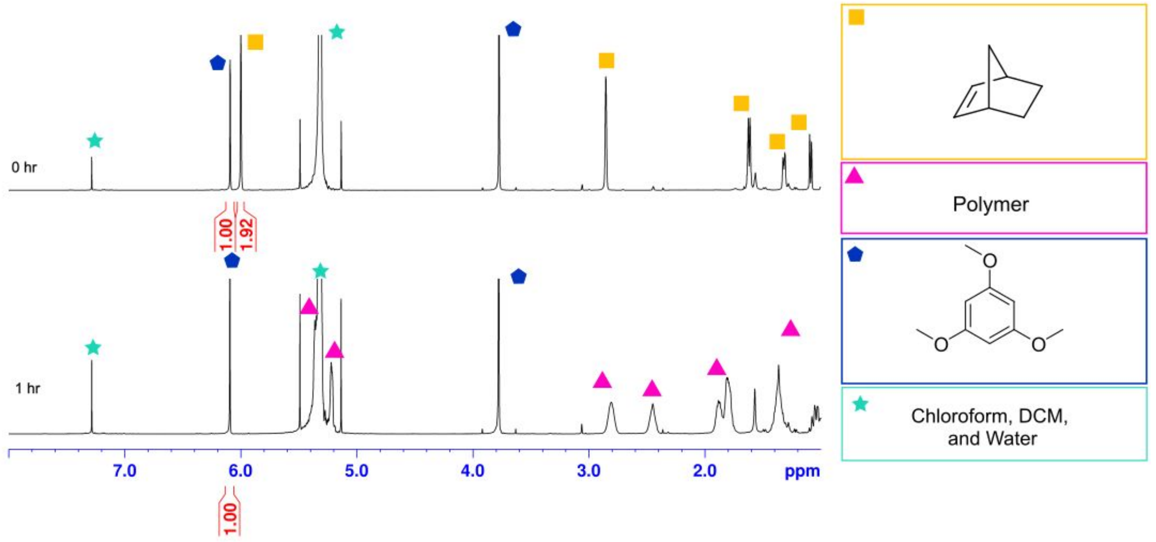

Figure S19. ^{1}H NMR data of monomer Nb (r84) and the corresponding polymer.

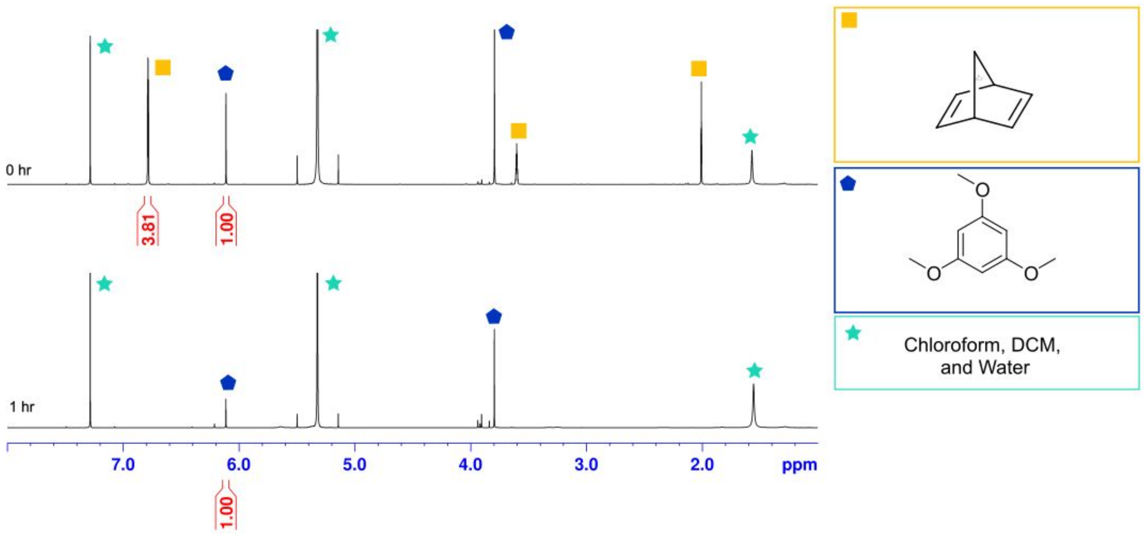

Figure S20. ^{1}H NMR data of monomer NbO (r85). The polymer signals are not visible due to lack of solubility.

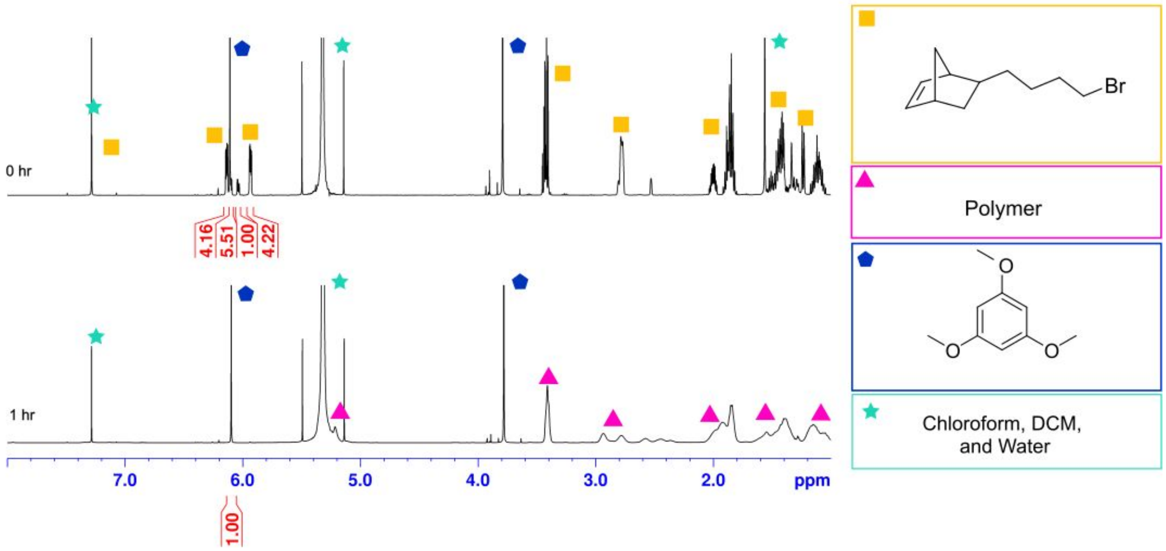

Figure S21. ^{1}H NMR data of monomer BrBuBb (r88) and the corresponding polymer.

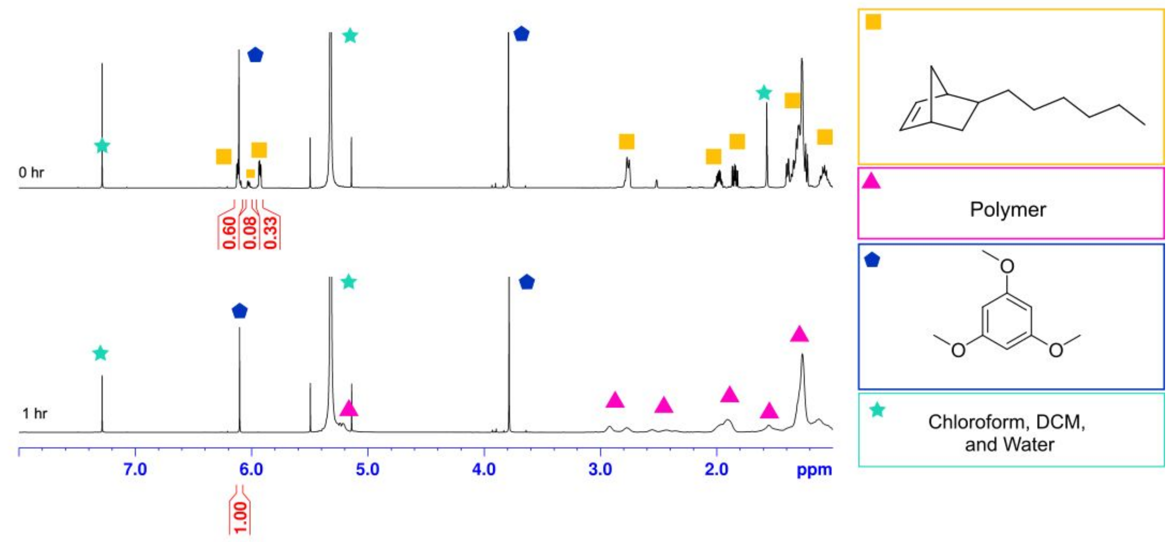

Figure S22. ^{1}H NMR data of monomer HxNb (r89) and the corresponding polymer.

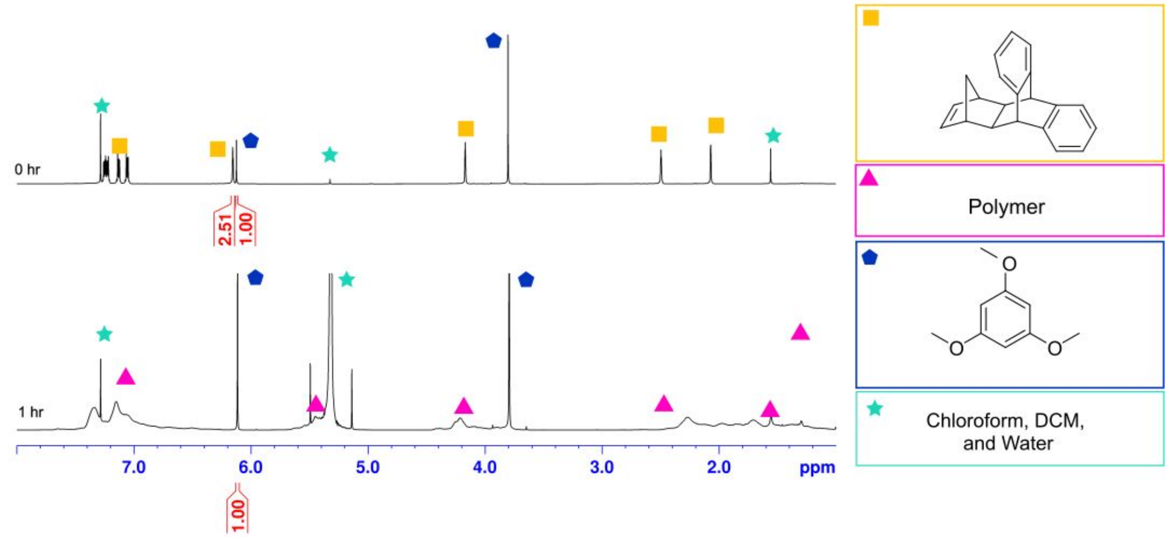

Figure S23. ^{1}H NMR data of monomer HbAn (r93) and the corresponding polymer.

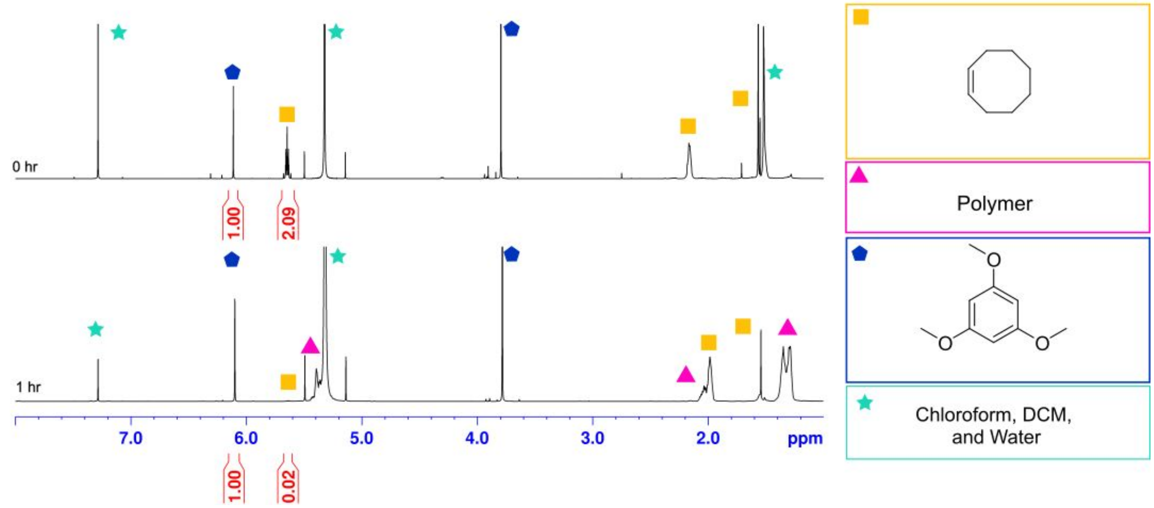

Figure S24. ^{1}H NMR data of monomer COE (r98) and the corresponding polymer.

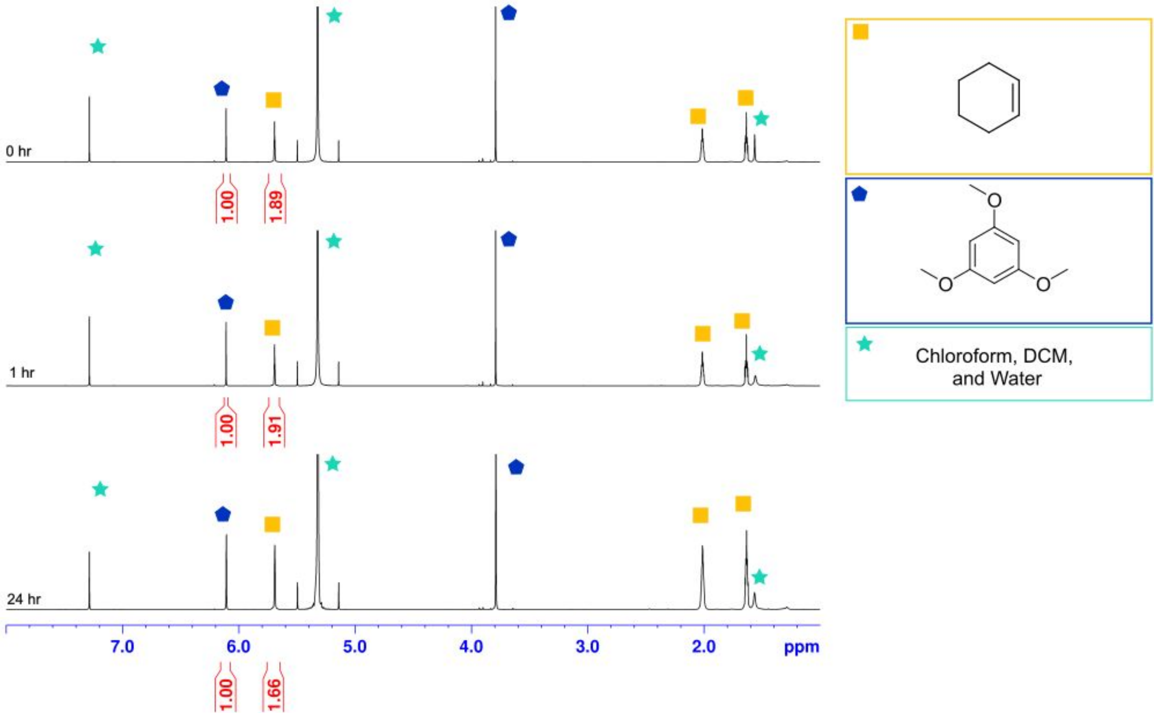

Figure S25. ^{1}H NMR data of monomer CHE (r101).

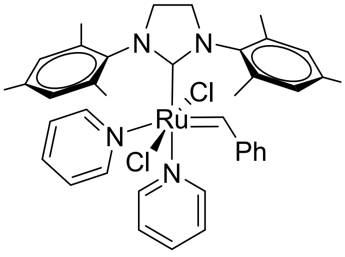

Figure S26. Grubbs third generation catalyst (G3).

Table S2. List of monomer full name and abbreviation

Monomer abbreviation	Monomer Full Name	Monomer used	
		(mg)	(mmol)
PhONDI (r2)	N-phenyloxanorbornenediimide	240	0.99
MPhONDI (r9)	1-methyl-N-phenyloxanorbornenediimide	255	1.00
DMPhONDI (r14)	1,4-dimethyl-N-phenyloxanorbornenediimide	267	0.99
DMONa (r75)	1,4-dimethyl-1,4-dihydro-1,4-epoxynapthalene	178	1.03
NbD (r85)	Norbornadiene	94	1.02
Nb (r84)	Norbornene	94	1.00
BrBuNb (r88)	Bromobutylnorbornene	230	1.00
CyONDI (r3)	N-cyclohexyloxanorbornenediimide	251	1.02
MCyONDI (r9)	1-methyl-N-cyclohexyloxanorbornenediimide	261	1.00
DMCyONDI (r15)	1,4-dimethyl-N-cyclohexyloxanorbornenediimide	275	1.00
NbAn (r93)	Norbornene-anthracene adduct	271	1.00
CHE (r101)	Cyclohexene	82	1.00
COE (r98)	Cyclooctene	112	1.02
HxNb (r89)	Hexylnorbornene	178	1.00

RSE for cage-like systems

To evaluate the performance on complex polycyclic systems, we computed the ring strain energies of prismane, cubane, and adamantane. Since the AIMNet2 RSE workflow is primarily designed for single-ring systems, some manual intervention was required. Each molecule was first processed to compute the RSE of one ring; the resulting structure was then re-submitted iteratively until all rings were accounted for. The total RSE was obtained by summing the strain released at each step. The workflow effectively automates RSE calculation for individual rings, while enabling extension to multi-ring systems through repeated application. Results are summarized in **Table S5** and show good agreement with reference values.

Table S3. RSE for prismane, cubane and adamantane

Name	Structure	RSE (kcal/mol)	Reference RSE (kcal/mol)
prismane	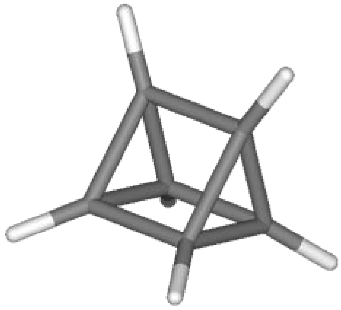	132.74	129.06 [Ref. [6]]
cubane	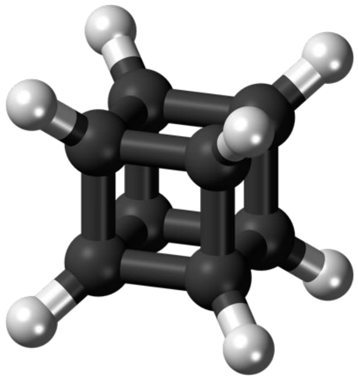	145.43	159.46 to 165 [Ref. [7,8]]
adamantane	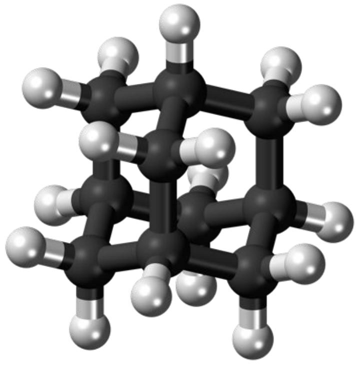	4.89	6.9 to 7.60 [Ref. [8,9]]

Reference

(1) Gordon, C. G.; MacKey, J. L.; Jewett, J. C.; Sletten, E. M.; Houk, K. N.; Bertozzi, C. R. Reactivity of Biarylazacyclooctynones in Copper-Free Click Chemistry. *J Am Chem Soc* **2012**, *134*, 9199–9208.

(2) Pierre, F.; Commarieu, B.; Tavares, A. C.; Claverie, J. High Tg Sulfonated Insertion Polynorbornene Ionomers Prepared by Catalytic Insertion Polymerization. *Polymer (Guildf)* **2016**, *86*, 91–97.

(3) Martínez-Arranz, S.; Alb́eniz, A. C.; Espinet, P. Versatile Route to Functionalized Vinylic Addition Polynorbornenes. *Macromolecules* **2010**, *43*, 7482–7487.

(4) Medina, J. M.; Ko, J. H.; Maynard, H. D.; Garg, N. K. Expanding the ROMP Toolbox: Synthesis of Air-Stable Benzonorbornadiene Polymers by Aryne Chemistry. *Macromolecules* **2017**, *50*, 580–586.

(5) Li, W.; Zhan, Q.; Yang, P. Synthesis of Poly(Maleimide)s with Promising Performance via Diels–Alder Reaction and Ring-Opening Metathesis Polymerization. *Journal of Polymer Research* **2023**, *30*, 127.

(6) Zhu, C.; Eckhardt, A. K.; Chandra, S.; Turner, A. M.; Schreiner, P. R.; Kaiser, R. I. Identification of a Prismatic P3N3 Molecule Formed from Electron Irradiated Phosphine-Nitrogen Ices. *Nat Commun* **2021**, *12*, 5467.

(7) Agapito, F.; Santos, R. C.; Borges Dos Santos, R. M.; Martinho Simões, J. A. The Thermochemistry of Cubane 50 Years after Its Synthesis: A High-Level Theoretical Study of Cubane and Its Derivatives. *Journal of Physical Chemistry A* **2015**, *119*, 2998–3007.

(8) Schreiner, P. R.; Fokin, A. A.; Šekutor, M. *The Chemistry of Diamondoids*; Wiley, 2024.

(9) Komarov, I. V. Organic Molecules with Abnormal Geometric Parameters. *Russian Chemical Reviews* **2001**, *70*, 991–1016.
