# Supplementary figures and images for: Fast and Accurate Ring Strain Energy Predictions with Machine Learning and Application in Strain-Promoted Reactions
Source: JACS Au. 2025 Oct 13;5(10):4750–61. doi: 10.1021/jacsau.5c00667 (PMC12569667; doi:10.1021/jacsau.5c00667)

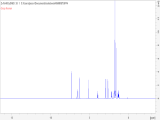

Supplement: Supplementary file 2 [file au5c00667_si_002.zip › H-NMR-Ringstrain/Z-JV-a-MCyONDI/20/pdata/1/thumb.png]

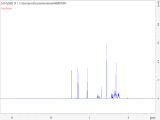

Supplement: Supplementary file 2 [file au5c00667_si_002.zip › H-NMR-Ringstrain/Z-JV-CyONDI/20/pdata/1/thumb.png]

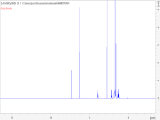

Supplement: Supplementary file 2 [file au5c00667_si_002.zip › H-NMR-Ringstrain/Z-JV-DMCyONDI/20/pdata/1/thumb.png]

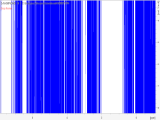

Supplement: Supplementary file 2 [file au5c00667_si_002.zip › H-NMR-Ringstrain/Z-JV-DMPhONDI/10/pdata/1/thumb.png]

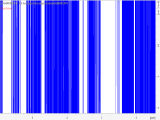

Supplement: Supplementary file 2 [file au5c00667_si_002.zip › H-NMR-Ringstrain/Z-JV-MPhONDI/10/pdata/1/thumb.png]

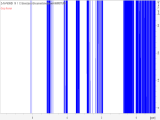

Supplement: Supplementary file 2 [file au5c00667_si_002.zip › H-NMR-Ringstrain/Z-JV-PhONDI/10/pdata/1/thumb.png]
